# Long‐Term Population Monitoring Reveals Changes in Mesocarnivore Occupancy in Response to Severe Drought

**DOI:** 10.1002/ece3.73960

**Published:** 2026-07-16

**Authors:** Jody Tucker, Marie Martin, David Green, Sean Matthews

**Affiliations:** ^1^ USDA Forest Service Rocky Mountain Research Station Missoula Montana USA; ^2^ Institute for Natural Resources Oregon State University Corvallis Oregon USA

**Keywords:** climate change, drought, fisher, gray fox, marten, mesocarnivore, occupancy modeling, ringtail

## Abstract

Rapid shifts in climate conditions can disrupt disturbance regimes and generate novel spatial conditions that have consequences for wildlife. In the montane Sierra Nevada range of California, USA, many species occur in sympatry, with local variation in elevation, climate conditions, and forest structure allowing species that may compete with each other to persist. However, shifting climate conditions in this region have potential impacts for species persistence and coexistence. From 2012 to 2015, a historic drought in California resulted in < 5% of expected snowpack and atypically warm winter conditions. These climatic conditions were thought to impact mesocarnivores, with particularly negative effects for species occurring at their range limits. Understanding how rapidly shifting conditions may affect species occurrence and persistence provides an opportunity to guide contemporary conservation efforts and better understand how continued change may imperil sensitive species. We examined the effects of this historic drought and contemporary forest structure on the occurrence of four sympatric mesocarnivores: Pacific martens ( *Martes caurina* ), fishers ( *Pekania pennanti* ), gray foxes ( *Urocyon cinereoargenteus* ), and ringtails ( *Bassariscus astutus* ). We used long‐term monitoring data in a dynamic spatial occupancy framework to identify the consequences of a historic drought event on the occurrence of these mesocarnivores and predict the effects of continued climate change on their persistence. We found that climate‐sensitive martens and fishers, who occur at the warm edges of their ranges within this system, generally exhibited declines in occupancy following warmer, drier years while gray foxes and ringtails thrived in these atypical conditions. Ringtails were the only species predicted to benefit from future changes in climate conditions, while fishers, gray foxes, and martens were predicted to decline under all climate change scenarios. Our work demonstrates the importance of identifying species‐specific responses to change and the potential consequences of climate change for sympatric communities.

## Graphical abstract

We examined the effects of a historic drought and contemporary forest structure on the occurrence of four sympatric mesocarnivores: Pacific martens ( *Martes caurina* ), fishers ( *Pekania pennanti* ), gray foxes ( *Urocyon cinereoargenteus* ), and ringtails ( *Bassariscus astutus* ). We used long‐term monitoring data in a dynamic spatial occupancy framework to identify the consequences of the drought on the occurrence of these mesocarnivores and predict the effects of continued climate change on their persistence.

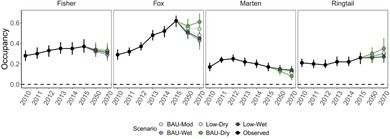

## 1 Introduction

Carnivores play a critical role in ecosystems including top‐down effects on regulating the lower tropic levels and creating fear‐driven direct and indirect effects on communities (Beschta and Ripple 2009; Laundré et al. 2010). Research on mammals is often biased toward larger‐bodied species, potentially due in part to both their disproportionate effects on ecosystem function and in part to their charisma and conspicuousness (dos Santos et al. 2020). Despite the attention that large, apex carnivores (e.g., mountain lions [ *Puma concolor* ], wolves [ *Canis lupus* ]) receive, the majority of carnivores are small or midsized (~< 15 kg) and are termed “mesocarnivores” (Prugh et al. 2009; Roemer et al. 2009). Mesocarnivores have been shown to be both top‐down and bottom‐up drivers of ecosystem function, either as an apex predator where other larger carnivores are absent or extirpated (Prugh et al. 2009), or as a mid‐trophic link that can influence ecosystems by regulating abundance or distribution of prey populations, dispersing seeds and spores, and altering impacts on primary producers (Marneweck et al. 2021).

Understanding the factors that shape mesocarnivore community ecology, whether abiotic (e.g., topography, climate) or biotic (e.g., habitat or species interactions), is important to be able to predict how the community may respond to changes in ecological conditions. Understanding the dynamics of ecological communities at species' warm‐edge range limits like their lower elevation or lower latitude regions may provide critical insights into how the species and communities adapt to warming global temperatures (Rehm et al. 2015). These warm‐edge margins are thought to be critical for long‐term species persistence because species there may possess local genetic adaptations that could help them adapt to warming temperatures (Lesica and Allendorf 1995; Rehm et al. 2015). While climate is considered an abiotic factor, past research has shown that biotic interactions also consistently influence warm‐edge boundaries (Paquette and Hargreaves 2021).

In the southern Sierra Nevada mountains of California, two mesocarnivore species of conservation concern, the Pacific fisher ( *Pekania pennanti* , hereafter fisher) and Pacific marten ( *Martes caurina* , hereafter marten), occur at their warm‐edge range limits (Zielinski et al. 2017). Fishers in the southern Sierra Nevada are listed as a federally endangered distinct population segment (U.S. Fish and Wildlife Service 2020) and marten are designated as a sensitive species by the U.S. Forest Service (U.S. Forest Service Pacific Southwest Region 2013). Both species are associated with forested ecosystems with late‐seral structural components (i.e., large trees, standing‐dead trees, multilayer canopy, coarse woody debris (Buskirk and Powell 1994, Green et al. 2019, Tweedy et al. 2019)) but exhibit different climatic associations. Martens are generally associated with cooler, wetter, and snowier conditions while fishers, whose greater foot‐loading makes them less adept at moving through snow, are limited by snow and can occur in warmer and drier climates than marten (Zielinski et al. 2017; Pauli et al. 2022). Where fisher and marten overlap, they often compete with one another for food (Smith et al. 2023, 2024), and occasionally fishers have been documented to kill marten (Krohn et al. 1995; McCann et al. 2010).

In the southern Sierra Nevada, fisher and marten co‐occur with two other similar‐sized mesocarnivores, gray fox ( *Urocyon cinereoargenteus* ) and ringtail ( *Bassariscus astutus* ) (Green et al. 2018). While gray foxes are common and do not have special status, ringtails are considered a Species of Greatest Conservation Need by the state of California (California Department of Fish and Wildlife 2025). Gray foxes and ringtails are not at their warm edge range limits in the southern Sierra Nevada with distributions extending far to the south, including in a wide array of warmer and drier climates than fisher and marten appear to tolerate (Reid et al. 2016; Roemer et al. 2016). As a result, gray foxes (Green et al. 2022) and ringtails (Gundermann et al. 2023) can thrive in disturbed and forested landscapes while martens and fishers typically decline when forest loss occurs (Green et al. 2022; Woollard et al. 2024; Martin et al. 2025). Nonetheless, fishers' larger body size can result in them being a dominant species when conditions favor them over smaller‐bodied martens (Fisher et al. 2013; Manlick et al. 2017; Jensen and Humphries 2019), gray foxes, and ringtails (Green et al. 2018). Increasingly heterogeneous and dynamic conditions within these landscapes could shift this intraguild hierarchy and require an understanding of how these species respond to landscape change and novel conditions.

Between 2012 and 2015, California experienced a severe, prolonged, and escalating drought. During the final and most severe year of the drought, the southern Sierra Nevada only received 5% of the expected average annual snowfall (Office of Environmental Health Hazard Assessment 2022). Cumulatively, the 4‐year drought period from 2012 to 2015 has been estimated to be a one in 10,000‐year climate event (Asner et al. 2016; Robeson 2015). The cascading effects of this drought on forest composition, water resources, wildlife habitat, and wildlife populations are not yet fully understood. In this study we take advantage of a long‐term carnivore monitoring dataset in this region to assess how fluctuations in climate and vegetation over time affected the spatial occupancy of fishers, martens, gray foxes, and ringtails. We investigate how occupancy patterns of these mesocarnivores shifted from 2002 to 2015, including the historic California drought from 2012 to 2015, and use contemporary relationships between species' occurrence and spatial conditions to predict the potential effects of shifting climate conditions.

## 2 Methods

### 2.1 Study Area

**FIGURE 1:**
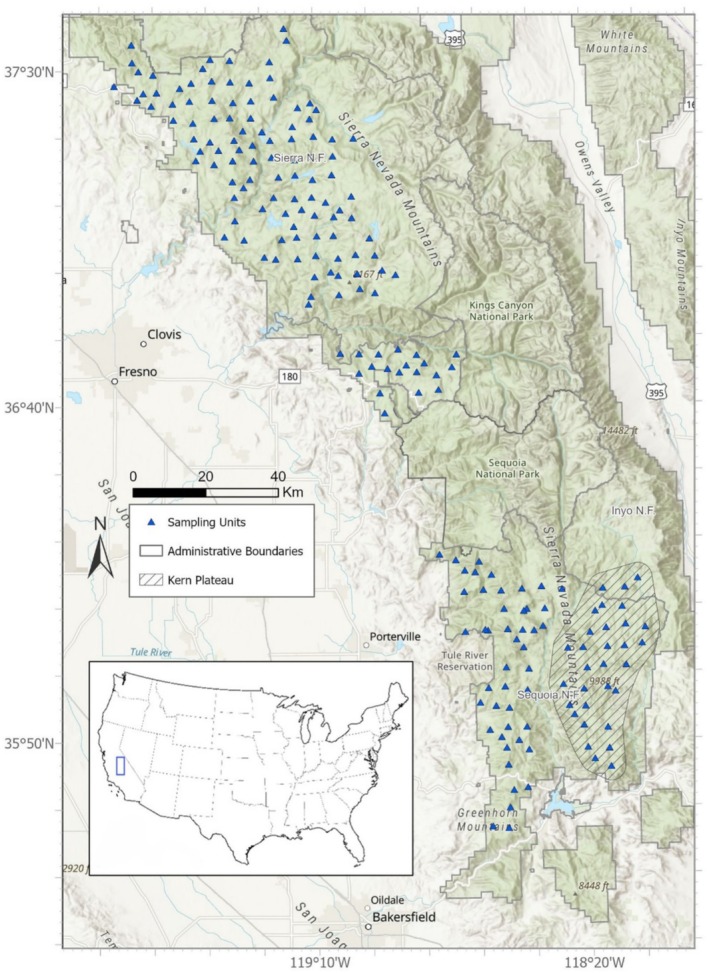
Locations of sampling unit centroids in the southern Sierra Nevada surveyed from 2002 to 2015.

Our study area encompassed the west slope of the Sierra Nevada mountains south of the Merced River on the U.S. Forest Service Sierra, Sequoia, and Inyo National Forests with some supplementary survey locations in Yosemite and Sequoia‐Kings Canyon National Parks (Figure 1). The Sierra Nevada has a Mediterranean climate with most precipitation occurring in the winter between October and May, falling primarily as snow at middle to high elevations (Cleland et al. 2007). The study area is also characterized by both elevational and precipitation gradients, with the landscape becoming both steeper and drier as you move southward. In the southern portion of the study area lies the Kern Plateau which is a high elevation plateau characterized by low annual precipitation (Zielinski et al. 2017).

### 2.2 Field Data Collection

Data was collected by the U.S. Forest Service Sierra Nevada Carnivore Monitoring Program from 2002 to 2015 (Zielinski et al. 2013; Tucker et al. 2014) which monitored sample units at fixed locations from May to October each year (Figure 1). The sampling frame consisted of 202 sampling units with a mean minimum distance between sample unit centroids of 4.1 km with an average of 122 units surveyed/year. Surveys consisted of repeated visits to sample units, which were comprised of arrays of three to six noninvasive sampling stations (trail cameras, track plates) spaced across an area of ~0.8 km^2^ with the centroid of this aray centered around a Forest Inventory and Analysis point (Zielinski et al. 2013, 2017). Both cameras and track plates were baited with meat and a scent lure (Gusto, Minnesota Trapline Products). From 2002 to 2009 (Phase 1), units consisted of six enclosed, ground level track plate stations that were spaced 500 m apart and checked five times over a 10‐day period. There was no sampling in 2010 due to funding and logistical limitations. In 2011 through 2015 (Phase 2), the survey protocol was revised to three pairs of stations 500–800 m apart, with each pair consisting of a track plate box as described above and trail camera 100 m apart. Baited trail cameras were deployed at defined monitoring locations a minimum of 50 m away from a road or trail. Trail cameras model varied across varied both within and between years and included two camera models, the Bushnell Trophy Cam and Trophy Cam HD. Phase 2 units were checked weekly over a 21‐day period resulting in a detection history of three checks. For more details regarding the study area, sample design, and noninvasive survey methods see Zielinski et al. (2013) and Tucker et al. (2014, 2024).

### 2.3 Spatial Covariates

We included two vegetation covariates: canopy cover and standard deviation of canopy cover (representing variation in canopy cover), found to be important component of fisher habitat (Purcell et al. 2009). Annual canopy cover data was obtained from LANDFIRE (https://www.landfire.gov/cc.php). For sampling years in between LANDFIRE updates we used the closest prior year of LANDFIRE data and then adjusted for possible major sources of canopy disturbances due to wildfire (Supporting Information). We included three climate variables previously found to influence fisher occurrence in the southern Sierra Nevada: total annual precipitation, April 1 snowpack, and average annual minimum temperature (Zielinski et al. 2017). Climate data was obtained from the 2014 California Basin Characterization Model (BCM) using 30‐year statistical summaries from 1981 to 2010 (Thorne et al. 2012; Flint et al. 2013, http://climate.calcommons.org/dataset/2014‐CA‐BCM). We rescaled spatial covariates from their native resolution (Table S1) using a the mean of a square moving window in the focal statistics tool in ArcGIS Desktop 10.7.1 (ESRI 2019) at two scales: (1) detection probability = 0.01 km^2^ (100 m × 100 m) around each sampling device and (2) occupancy = 6.25 km^2^ (2.5 km × 2.5 km, ~female fisher home range size; Zielinski et al. 2004).

### 2.4 Dynamic Occupancy Model

We used a spatial dynamic occupancy model to estimate the detection probability, occupancy, persistence, and colonization of fishers, gray foxes, ringtails, and martens, from 2002 to 2015. We delineated the model state‐space by generating a minimum convex polygon encompassing all sampling units and subsequently split the state‐space into 2500 m × 2500 m grid cells. Our model state‐space encompassed two regions: (1) the Sierra and Sequoia National Forests (i.e., “Sierra”), where conditions are cooler, wetter, and all four species of interest occurred, and (2) the Kern Plateau, which occurs within the Sequoia and Inyo National Forests, where conditions are drier, and martens and ringtails were generally absent (i.e., < 14 detections during 14‐year study period).

#### 2.4.1 Detection Probability

We modeled the observation process (*y*_*u,t,r,w,s*_; detection of species *s* at station *r* in unit *u* in week *w* in year *t* where *y*_*u,t,r,w,s*_ = 1 when species *s* was detected and zero otherwise) as a Bernoulli‐distributed variable with probability equal to the product of true occupancy of the species of interest (*z*_*u,t,r,w,s*_) and detection probability of the species of interest (*p*_*u,t,r,w,s*_), so that detection probability was conditional on the species being present. We further modeled detection probability accounting for station‐ and survey‐specific covariates as fixed effects using a logit‐link function (logit(*p*_*u,t,r,w,s*_) = β_0*s,t*_ + β1_*s*_ * *x*_*1u,r,t*_ + … + β_n_ *x*_*n*_, where β represents the coefficient of detection predictors and *x* represents the detection predictor). We allowed detection intercepts (β_0_ for trackplates, c_0_ for cameras) to vary by year and included canopy cover, standard deviation of canopy cover, and prior detection at station *r* as predictors on detection probability at both track plates and cameras and further included camera models as predictors on detection probability at cameras. These covariates allowed us to evaluate the effects of fine‐scale forest cover (canopy cover) and heterogeneity (standard deviation of canopy cover) and evaluate whether species were more likely to be detected at stations where they were previously detected.

We used the following equations to model the observation process for each species *s* detected by track plates:

$$y_{u,t,r,w,s}^{\text{trackplate}}\sim \text{Bernoulli}\left(\left[z_{u,t,r,w,s}*p_{u,t,r,w,s}^{\text{trackplate}}\right]\right)$$

$$\begin{array}{cc} \text{logit}\left(p_{u,t,r,w,s}^{\text{trackplate}}\right)= & \beta_{\text{0t},s}+\beta_{\text{1s}}*{\text{canopy cover}}_{u,r,t}+\beta_{\text{2s}}*\mathrm{sd}\;{\text{canopy cover}}_{u,r,t}\\ & +\beta_{\text{3s}}*\text{previously detected}\;\mathrm{at}\;{\text{trackplate}}_{u,t,r,w,s} \end{array}$$

We used the following equations to model the observation process for each species *s* detected by remote cameras:

$$y_{u,t,r,w,s}^{\text{camera}}\sim \text{Bernoulli}\left(\left[z_{u,t,r,w,s}*p_{u,t,r,w,s}^{\text{camera}}\right]\right)$$

$$\begin{array}{cc} \text{logit}\left(p_{u,t,r,w,s}^{\text{camera}}\right)= & c_{\text{0t},s}+c_{\text{1s}}*{\text{canopy cover}}_{u,r,t}\\ & +c_{\text{2s}}*\mathrm{sd}\;{\text{canopy cover}}_{u,r,t}\\ & +c_{\text{3s}}*\text{cameramodel1}\\ & +c_{\text{4s}}*\text{cameramodel2}\\ & +c_{\text{5s}}*\text{previously detected}\;\mathrm{at}\;{\text{camera}}_{u,t,r,w,s} \end{array}$$

We defined statistical significance for model covariates for both detection and occupancy (subsequent section) as a covariate having a ±95% credible interval that did not overlap zero.

#### 2.4.2 Occupancy

We modeled initial occupancy in year 1 for each species *s* (*z*_*gs1*_ = 1 if species *s* occurred in grid cell *g* and *z*_*gs1*_ = 0 if not) as a latent random variable with a Bernoulli distribution with occupancy probability 𝜓_*gs1*_ (i.e., probability that species *s* occurred in grid cell *g*) as a function of spatial covariates via a logit‐link function (logit(𝜓_*gs1*_) = α_0s_ + α_1s_ *x*_1*g1*_ + … + α_ns_ *x*_n*g1*_, where α represents coefficients of the spatial covariates for each species *s* and *x* represents the value of the spatial covariate of interest at grid cell *g*):

$$z_{g,s,1}\sim \text{Bernoulli}\left({\text{𝜓}}_{g,s,1}\right)$$

$$\begin{array}{cc} \text{logit}\left({\text{𝜓}}_{g,s,1}\right)= & \alpha_{\text{0s}}+{\alpha_{\text{1s}}}^{*}\;{\text{canopy cover}}_{g,1}\\ & +{\alpha_{\text{2s}}}^{*}\;\mathrm{sd}\;{\text{canopy cover}}_{g,1}\\ & +{\alpha_{\text{3s}}}^{*}\;{\text{snow depth}}_{g,1}\\ & +{\alpha_{\text{4s}}}^{*}\;{\text{precipitation}}_{g,1}\\ & +{\alpha_{\text{5s}}}^{*}\;{\text{minimum temperature}}_{g,1} \end{array}$$

We modeled occupancy for each subsequent year *t* for each species *s* (*z*_*gst*_ = 1 if species *s* occurred at grid cell *g* in year *t* and *z*_*gst*_ = 0 if not) where occupancy probability 𝜓_*gst*_ (i.e., probability that species *s* occurred at grid cell *g* in year *t*) is influenced by persistence (i.e., Φ_*gst*_, the probability of grid cell *g* being occupied by species *s* in the previous year *t−1* remaining occupied in the following year *t*) and colonization (i.e., γ_*gst*_, the probability of grid cell *g* being unoccupied by species *s* in the previous year *t−1* becoming occupied in the following year *t*). We estimated persistence and colonization as a function of spatial covariates via a logit‐link function (i.e., logit(Φ_*gst*_) or logit(γ_*gst*_) = α_0s_ + α_1s_ *x*_1*gt*_ + … + α_ns_ *x*_n*gt*_, where α represents coefficients of the spatial covariates [i.e., occurrence predictor] for each species *s* and *x* represents the value of the spatial covariate of interest at grid cell *g*).

We used the following equations to model occupancy, persistence, and colonization in each subsequent year *t* for each of four carnivore species *s* in each grid cell *g*:

$$z_{g,t,s}\sim \text{Bernoulli}\left(\left[{z_{g,t-1,s}}^{*}\;\Phi_{g,t,s}\right]+\left[1-{z_{g,t-1,s}}^{*}\;\gamma_{g,t,s}\right]\right)$$

$$\begin{array}{cc} \text{logit}\left(\Phi_{g,t,s}\right)= & \Phi_{\text{0s}}+{\Phi_{\text{1s}}}^{*}\;{\text{canopy cover}}_{g,t}\\ & +{\Phi_{\text{2s}}}^{*}\;\mathrm{sd}\;{\text{canopy cover}}_{g,t}\\ & +{\Phi_{\text{3s}}}^{*}\;{\text{snow depth}}_{g,t}\\ & +{\Phi_{\text{4s}}}^{*}\;{\text{precipitation}}_{g,t}\\ & +{\Phi_{\text{5s}}}^{*}\;{\text{minimum temperature}}_{g,t} \end{array}$$

$$\begin{array}{cc} \text{logit}\left(\gamma_{g,t,s}\right)= & \gamma_{\text{0s}}+{\gamma_{\text{1s}}}^{*}\;{\text{canopy cover}}_{g,t}\\ & +{\gamma_{\text{2s}}}^{*}\;\mathrm{sd}\;{\text{canopy cover}}_{g,t}\\ & +{\gamma_{\text{3s}}}^{*}\;{\text{snow depth}}_{g,t}\\ & +{\gamma_{\text{4s}}}^{*}\;{\text{precipitation}}_{g,t}\\ & +{\gamma_{\text{5s}}}^{*}\;{\text{minimum temperature}}_{g,t} \end{array}$$

### 2.5 Predicted Responses to Climate Change

To predict how continued changes in climate may affect the occupancy of fishers, foxes, martens, and ringtails in the future, we obtained 30‐year statistical summaries from the BCM data for 5 climate change models for two future time periods: (1) 2040–2069, and (2) 2070–2099 (Flint et al. 2013). Three models represent business‐as‐usual scenarios with no emissions reductions from current levels and warmer temperatures across a range of projected precipitation changes (CNRM_rcp85: wetter, CCSM4_rcp85: moderate, MIROC_rcp85: drier). Two models represent mitigated reduced emission scenarios that have smaller projected temperature increases than the business‐as‐usual models (MPI_rcp45: wetter, MIROC_rcp45: drier). Overall, these reflect a broad range of emissions scenarios (Vermeulen 2014, L. Flint pers. comm). We then used the final year of our occupancy estimates (2015) to predict how occupancy in each grid cell might change given predicted changes in climate covariates. For each future time period *f* and each climate projection scenario *x*, we repeated our dynamic occupancy model to estimate predicted occupancy in each grid cell *g* for each species *s* as a product of spatial covariates:

$$z_{f,g,s,x}\sim \text{Bernoulli}\left(\left[{z_{g,2015,s}}^{*}\;\Phi_{f,g,t,x}\right]+\left[1-{z_{g,2015,s}}^{*}\;\gamma_{f,g,t,x}\right]\right)$$

$$\begin{array}{cc} \text{logit}\left(\Phi_{f,g,s,x}\right)= & \Phi_{\text{0s}}+{\Phi_{\text{1s}}}^{*}\;{\text{canopy cover}}_{g,x}\\ & +{\Phi_{\text{2s}}}^{*}\;\mathrm{sd}\;{\text{canopy cover}}_{g,x}\\ & +{\Phi_{\text{3s}}}^{*}\;{\text{snow depth}}_{g,x}\\ & +{\Phi_{\text{4s}}}^{*}\;{\text{precipitation}}_{g,x}\\ & +{\Phi_{\text{5s}}}^{*}\;{\text{minimum temperature}}_{g,x} \end{array}$$

$$\begin{array}{cc} \text{logit}\left(\gamma_{f,g,s,x}\right)= & \gamma_{\text{0s}}+{\gamma_{\text{1s}}}^{*}\;{\text{canopy cover}}_{g,x}\\ & +{\gamma_{\text{2s}}}^{*}\;\mathrm{sd}\;{\text{canopy cover}}_{g,x}\\ & +{\gamma_{\text{3s}}}^{*}\;{\text{snow depth}}_{g,x}\\ & +{\gamma_{\text{4s}}}^{*}\;{\text{precipitation}}_{g,x}\\ & +{\gamma_{\text{5s}}}^{*}\;{\text{minimum temperature}}_{g,x} \end{array}$$

### 2.6 Model Fitting and Assessment

We fit our occupancy model in Program R v. 4.0.5 (R Core Team 2021) using the Markov chain Monte Carlo (MCMC) methods of JAGS v. 4.2.0 (Plummer 2003). We calculated parameter estimates from 15,000 MCMC samples, taken from 3 chains run for 50,000 iterations, thinned by 10, following a burn‐in of 100,000. We assessed model convergence by examining trace plots and R values for convergence (Gelman and Hill 2006; Gelman et al. 2013). We present the medians and 95% Credible Intervals (CI) for our results.

## 3 Results

### 3.1 Data Collection and Detection Probability

**FIGURE 2:**
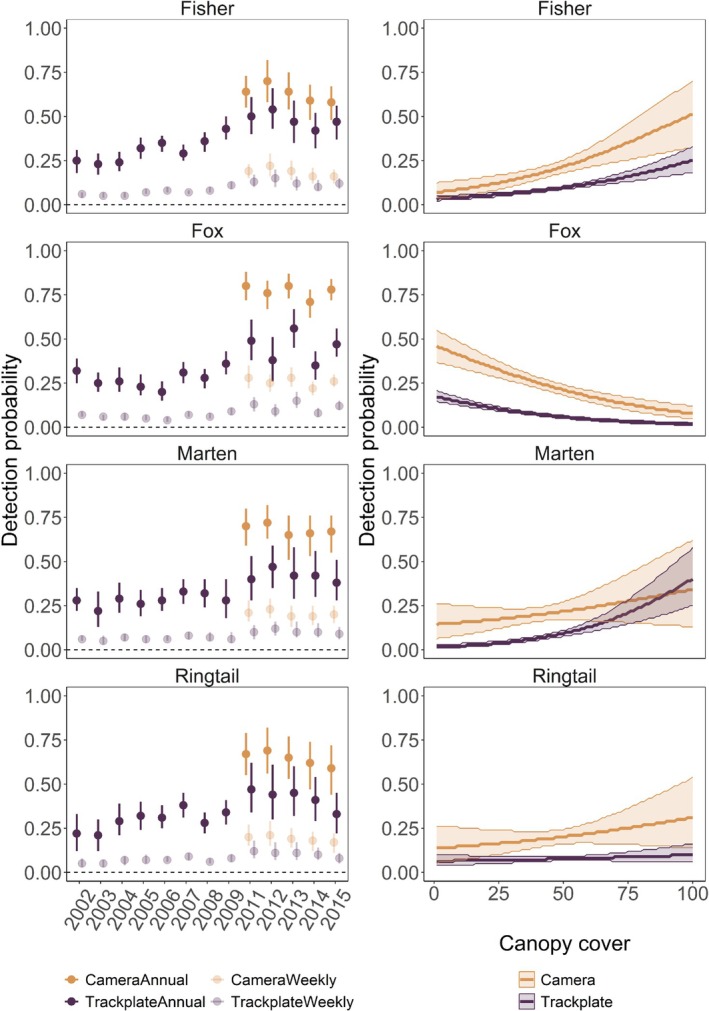
Detection probabilities and the effect of canopy cover on detection probability for fishers, gray foxes, martens, and ringtails within the study area in the Sierra Nevada, California, USA. The left panels indicate detection probability during each sampling season with solid dots (+ lines) representing mean annual detection probability estimates (±95% CIs) and transparent dots (+ lines) represent mean weekly detection probability estimates (±95% CIs) at cameras (purple) and track plates (green).

We sampled an average of 121.9 sample units annually (SD = 29.8). Species‐specific detection probability was generally stable during the study, with slightly higher detection probability for all species beginning in 2011 when we began using both cameras and track plates. Detection probabilities differed between sampling devices, with cameras exhibiting higher detection probability than track plates for all species and years. Detection probabilities were similar among fishers, martens and ringtails, but gray fox generally exhibited higher detection probability, which was particularly notable at cameras during phase 2 (mean annual $p^{\text{camera}}$(SD) 2011–2015: fisher = 0.60 [0.04], marten = 0.66 [0.04], ringtail = 0.61 [0.04], fox = 0.76 [0.03]) (Table S2, Table S3, Figure 2).

### 3.2 Covariate Relationships

#### 3.2.1 Detection Probability

Vegetation structure had differing effects on species‐specific detection probability. Detection probability of fishers at both cameras and track plates and martens at track plates was higher in areas with greater canopy cover, while fox detection probability was negatively related to canopy cover for both devices. Increasing variation in canopy cover was associated with higher detection probability of ringtails and lower detection probability of martens at cameras and track plates and was negatively related to fisher detection probability at cameras (Table S4). All four species were more likely at both station types to be detected where the species was detected previously. Camera model did not strongly affect detection probability of any species (Table S4).

#### 3.2.2 Initial Occupancy

**FIGURE 3:**
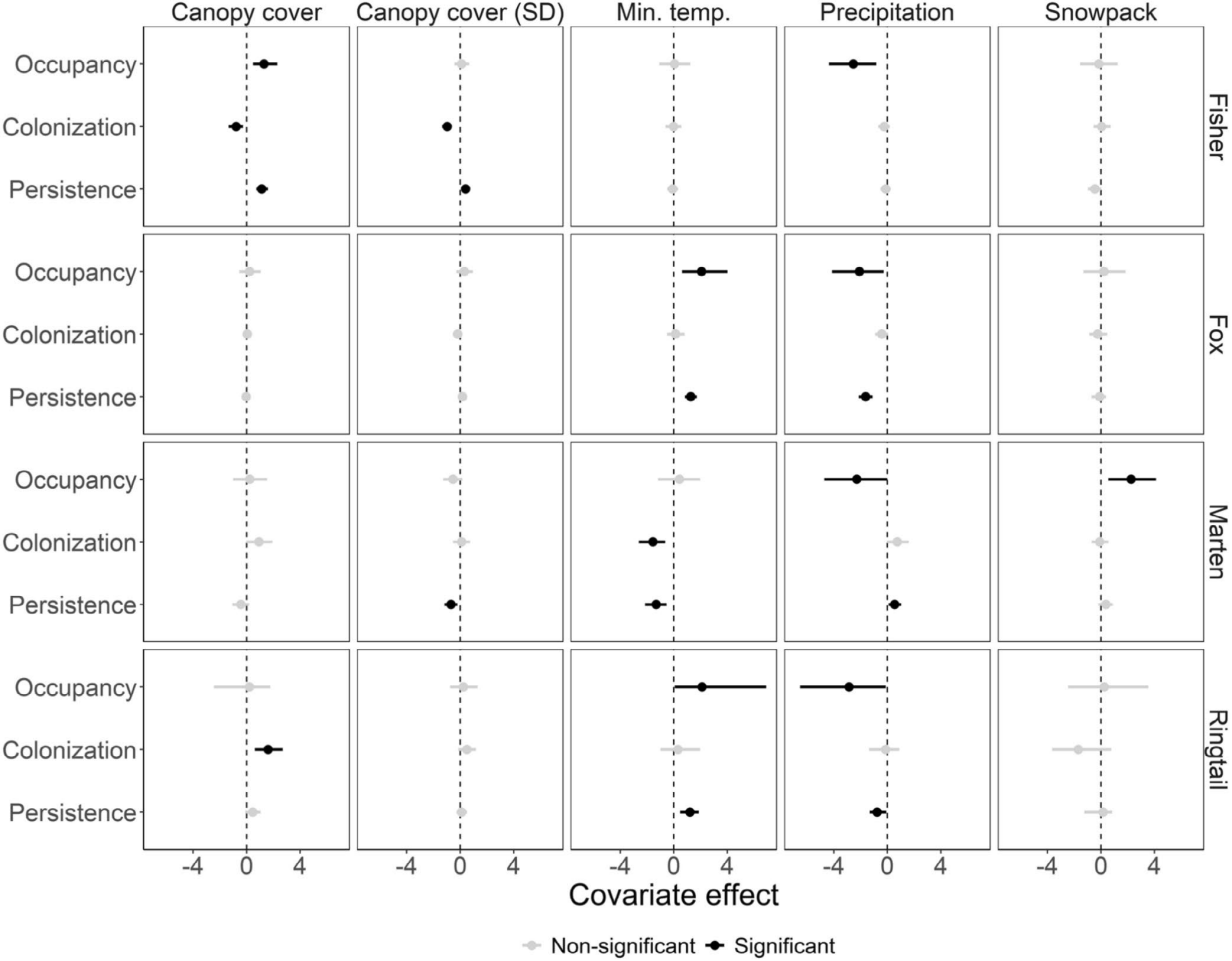
Effects of forest and climate covariates on initial occupancy (i.e., year 1 [2002]), probability of persistence (i.e., occupied site remains occupied), and probability of colonization (i.e., unoccupied site becomes occupied) for fishers, gray foxes, martens, and ringtails within the study area in the Sierra Nevada, California, USA. Circles (±bars) represent the mean effect (±95% credible intervals) of the covariate of interest, with black indicating effects that do not overlap zero and gray indicating effects that do overlap zero.

Fishers were the only species that showed a significant relationship with a vegetation covariate. Initial occupancy of fishers was higher in areas with greater canopy cover, but canopy cover had no effect on initial occupancy of foxes, martens, or ringtails, and standard deviation of canopy cover had no effect on initial occupancy of any species. Alternatively, all four species showed significant relationships with one or more climate covariates. Both fox and ringtail occupancy were positively influenced by higher minimum temperatures and negatively influenced by increasing precipitation. Fisher and marten occupancy were both negatively associated with increasing precipitation, with marten occupancy also positively associated with increasing snowpack (Figure 3, Table S5).

#### 3.2.3 Colonization and Persistence

Canopy cover had varied effects on the colonization and persistence of the carnivore guild—fishers were less likely to colonize but more likely to persist at sites with higher canopy cover. Fisher persistence was also positively related to higher variation in canopy cover. Ringtail persistence was also positively affected by canopy cover, but canopy cover did not influence either colonization or persistence of martens and foxes. Climate covariates had strong effects on persistence and colonization of martens and foxes, but relationships varied between the species. Martens were less likely to colonize and persist in warmer areas, while foxes and ringtails were more likely to persist in warmer areas. Increased precipitation positively influenced marten persistence but negatively influenced fox and ringtail persistence (Figure 3, Table S5).

### 3.3 Occupancy Patterns

**FIGURE 4:**
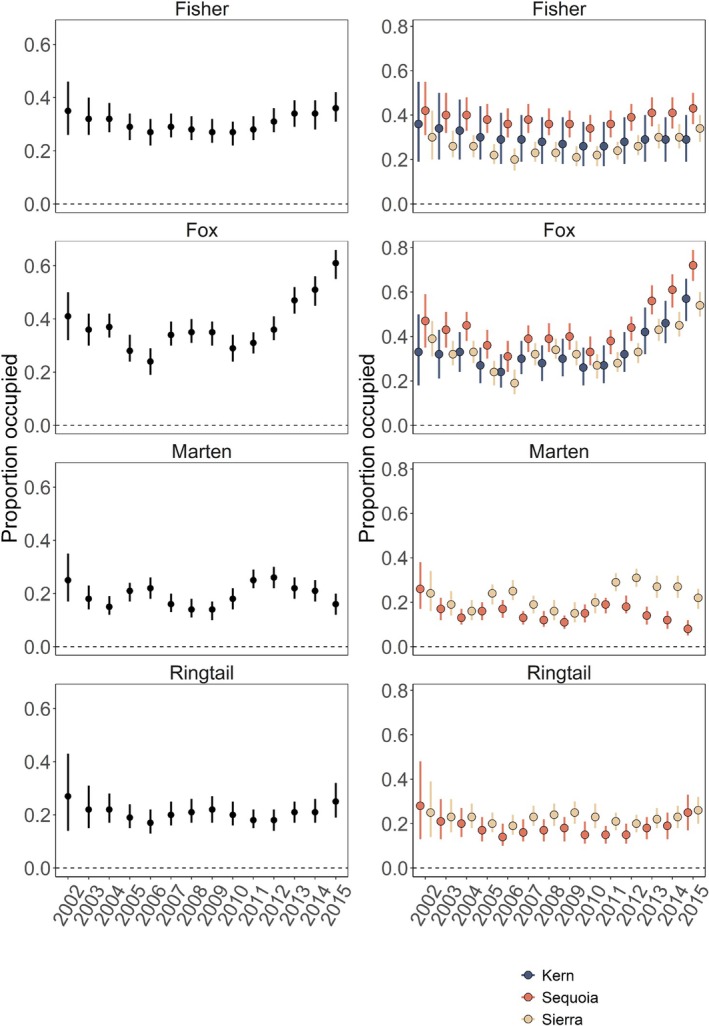
Species‐specific annual occupancy (mean, ±95% credible intervals) estimates within the entire study area (left panels) and by subregions (right panels) for fishers, gray foxes, martens, and ringtails within the study area in the Sierra Nevada, California, USA.

Fisher occupancy was relatively stable over time (𝜓_2002_ = 0.39 [0.29–0.54], 𝜓_2015_ = 0.37 [0.31–0.44]) with some variation between years (Table S6). Marten occupancy fluctuated, with highest occupancy in years following high winter snowpack (i.e., 𝜓_2006_ = 0.22 [0.18–0.25], 𝜓_2012_ = 0.25 [0.22–0.29]) that then declined in lower snowpack years in the middle and end of the study (Figure 4, Table S6). Ringtail occupancy increased steadily over time, showing a ~54% increase over the study period (𝜓_2002_ = 0.14 [0.07–0.23], 𝜓_2015_ = 0.26 [0.20–0.31]). Gray fox occupancy fluctuated from 2002 to 2012 but then increased rapidly as drought occurred (𝜓_2012_ = 0.37 [0.33–0.41], 𝜓_2015_ = 0.62 [0.56–0.67]). This reflects a ~70% increase in gray fox occupancy within 4 years (Figure 4).

### 3.4 Regional and Elevational Variation in Occupancy

**FIGURE 5:**
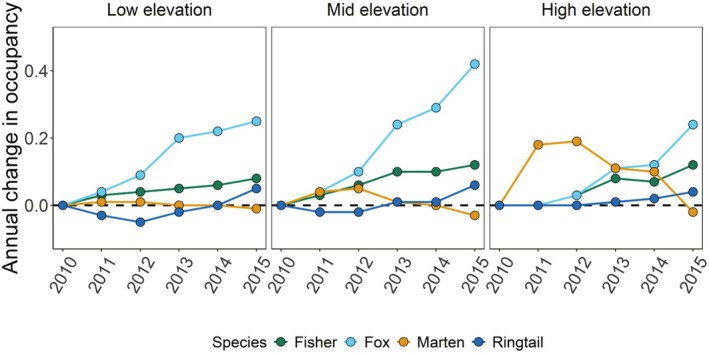
Species‐specific changes in annual occupancy for fishers, gray foxes, martens, and ringtails during a high snowpack period (2010–2011) and subsequent historic drought (2012–2015) within the study area in the Sierra Nevada, California, USA. Circles represent change in annual occupancy from baseline occupancy in 2010 with values above 0 indicating an increase in occupancy and values below zero indicating a decrease in occupancy.

Fishers and gray foxes exhibited similar occupancy patterns with higher occupancy on the Sequoia National Forest and lower occupancy on the Sierra National Forest. Conversely, martens and ringtails had lower occupancy on the Sequoia National Forest and higher on the Sierra National Forest until the end of the study period when occupancy rates for ringtail converged. Marten occupancy on the Sequoia National Forest declined ~70% from 2002–2015 (𝜓_2002_ = 0.24 [0.15–0.38], 𝜓_2015_ = 0.08 [0.05–0.12]) with majority of this decline during 2012–2015. However, marten occupancy on the Sierra National Forest remained relatively stable (𝜓_2002_ = 0.26 [0.17–0.35], 𝜓_2015_ = 0.22 [0.18–0.28]). Fisher occupancy was generally stable on the Sequoia National Forest but showed minor declines on the Kern Plateau over the 14‐year study period (23%: 𝜓_2002_ = 0.43 [0.26–0.61], 𝜓_2015_ = 0.33 [0.22–0.47]). Fisher on the Sierra National Forest during this period had oscillating occupancy patterns with lowest occupancy in 2005–2009. During the drought (2012–2015) gray fox occupancy strongly increased in at all elevations, while fisher and ringtail exhibited the largest increase at high elevations. Marten occupancy exhibited an oscillating pattern with occupancy highest in the sampling year after a winter with heavy snow accumulation (e.g., winters 2005–2006, 2011–2012) particularly at high elevations, but then rapidly declined during drought years (Figure 5, Table S8).

### 3.5 Climate Models

**FIGURE 6:**
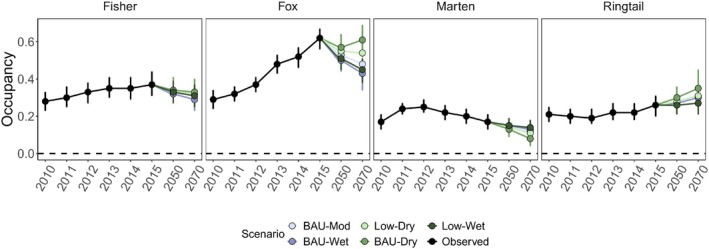
Observed (2010–2015) and predicted (2050–2070 and 2070–2100) species‐specific occupancy for fishers, gray foxes, martens, and ringtails under five climate projection scenarios (Business‐as‐usual emissions [BAU] moderate [CCSM4_rcp85], BAU wet [CNRM_rcp85], BAU dry [MIROC_rcp85], low emissions wet [MPI_rcp45], and low emissions dry [MIROC_rcp45]) and two future time periods within the study area in the Sierra Nevada, California, USA.

Despite increasing rapidly during drought years, fox occupancy was predicted to decline under most climate projection scenarios and time periods but remained stable in drier climate scenarios and above pre‐drought baseline occupancy estimates under all scenarios (Figure 6, Tables S6 and S7). Projected fisher and marten occupancy declined across most climate models (Figure 6), with fisher occupancy returning to approximately pre‐drought levels at low elevations but remaining higher at middle and high elevations (Figure S1). Conversely, marten occupancy shows a steady decline from the already depressed rates seen at the end of the drought, with further declines at high elevations where martens were most likely to occur. Predicted marten declines ranged from −4% to −23% by 2070–2099, with the strongest predicted declines occurring in drier climate scenarios. Ringtails were the only species predicted to benefit under projected climate scenarios, with occupancy predicted to increase during both time periods across all climate scenarios and the strongest increases predicted to occur under drier scenarios (Figure 6).

## 4 Discussion

Climate change is predicted to increase the frequency and magnitude of extreme weather events and anomalies (Meehl et al. 2000). Here, we show how extreme weather, in this case a multiyear high‐severity drought, can influence the wildlife community within an ecosystem and may have implications for their occurrence in the future. We found climate patterns influenced the occupancy of fishers, martens, gray foxes, and ringtails. Fox and ringtail occupancy increased during the drought period. These two species are adapted to warmer and drier conditions with distributions that extend much farther south than the study area. Fishers and martens, however, are two species that are at their warm‐edge limit of their distribution in the southern Sierra Nevada (Zielinski et al. 2017). While fisher occupancy was relatively stable during the study period, we estimated modest increases in occupancy during the drought period. Fishers are thought to be limited by deep snow (Krohn et al. 1995; Pauli et al. 2022), and this increase in occupancy could reflect fishers exploiting the loss of snowpack during the drought and occupying more middle and higher elevation areas during this period. Exhibiting spatial plasticity to take advantage of areas they do not typically occur in may have supported fisher resilience to the exceptionally dry conditions during the drought. Martens, on the other hand, are snow‐dependent and their occupancy declined at high elevations during the drought period when snow accumulation was historically low.

In this study, occupancy of all species was influenced by climate covariates, but only fisher occupancy was associated with vegetation. Fisher occupancy, colonization, and persistence were all strongly related to canopy cover and/or variation in canopy cover. Interestingly while initial occupancy and persistence were positively related to canopy cover, colonization had the opposite relationship with canopy cover as fisher colonization was higher at sites with lower canopy cover. While this negative relationship seems unexpected one possible explanation is if higher canopy cover sites were more likely to be occupied and stay occupied then the sites available for colonization would be those with lower canopy cover. While fishers are often considered a cold‐adapted species, this relationship with canopy cover highlights that managers and practitioners may be able to support fisher persistence in warmer and drier conditions by managing forests to increase tree persistence and resilience to drought (e.g., van Mantgem et al. 2016; Selwood et al. 2018). The importance of forest structure to fisher occupancy may also foreshadow future declines, as this drought resulted in widespread tree mortality and a dramatic increase in high severity wildfires that have altered the composition of forests and fisher habitat in the Sierra Nevada (Steel et al. 2023; Hart et al. 2025).

Using a dynamic occupancy modeling approach allowed us to derive annual occupancy patterns while accounting for interannual variation in persistence and colonization due to changes in annual environmental conditions (e.g., MacKenzie et al. 2003). While dynamic occupancy models have been proposed as an effective method for understanding distribution and range dynamics (Royle and Kéry 2007; Kéry et al. 2013), we do acknowledge there are potential limitations to our modeling approach. In particular, unmodelled sources of heterogeneity, including additional covariates or interspecific interactions that influence occupancy, persistence, and colonization, can affect parameter estimates and precision, particularly for uncommon species (MacKenzie et al. 2017), and remains an understudied aspect of dynamic occupancy models (Kelleher et al. 2026).

This mesocarnivore guild is known to exhibit competition for space and resources in the Sierra Nevada (Zielinski et al. 2017; Smith et al. 2024) and throughout other areas of sympatry (e.g., Green et al. 2018; Smith et al. 2023). Fishers have been hypothesized to limit gray fox abundance and distribution (Green et al. 2018) and are often thought to subordinate martens (Pauli et al. 2022). For example, research in northern California found that fisher removals during a translocation effort were associated with a subsequent increase in fox occupancy and that fishers limited the persistence of gray fox in areas where they co‐occurred (Green et al. 2018). Though these relationships can be agonistic, with fishers documented killing or consuming both gray foxes (McNeil et al. 2017; Pilgrim et al. 2023) and martens (McCann et al. 2010), intraguild competition of these species often manifests through resource exclusion (e.g., prey, habitat: Smith et al. 2023, Smith et al. 2024). The substantial increase in gray fox occupancy observed during the drought in this study may be an indirect indicator of an underlying reduction in competitor abundance or density, and potential for competitive release for foxes. Given occupancy and density do not exhibit a linear relationship, particularly for carnivores (Linden et al. 2017), we were not able to empirically demonstrate that declines in competitor abundance were a mechanism that facilitated rapid increases in gray fox occupancy. Future efforts to better understand the potential effects of shifting climate conditions on species occurrence and sympatry, particularly for low density, behaviorally cryptic carnivores, would benefit from incorporating methods that facilitate abundance estimates.

Similar to other studies, we found significant relationships between marten occupancy and snow conditions. The initial occupancy of martens was positively influenced by greater April 1 snowpack, but negatively influenced by increasing precipitation indicating that martens are less likely to occupy areas where precipitation falls mainly as rain or snow that does not persist and contribute to the snowpack. However, colonization and persistence were positively related to higher precipitation and lower minimum temperatures. This combination of high precipitation and low temperatures functions as a surrogate for winter snowfall as the majority of precipitation in the southern Sierras occurs during winter as snow. Our data indicates that overall marten population stability is driven by winters with heavy precipitation that causes periodic increases in occupancy that then declines in intervening years of average to below average snowfall. As observed during the drought and with predicted warming from climate change, without these years of heavy snow, marten occupancy continued to decline. In the snowier Sierra National Forest in the northern half of the study area, martens exhibited stable occupancy over time despite interannual variation in snowpack. However, in the warmer and drier Sequoia National Forest to the south, intermittent high occupancy during years with high snowpack (e.g., 2011–2012) were not enough to counteract the general decline in marten occupancy over time. Over time, with the warming predicted to occur in the coming decades it is likely that martens will be lost from the southernmost portions of the study area as its warm edge range limit contracts northward.

Future climate models for the Sierra Nevada generally predict increasing temperatures and decreasing snow accumulation, even in “wetter” climate scenarios (Dai 2013). Accordingly, fisher and marten occupancy is likely to decline under all climate scenarios, with marten declines strongest in business‐as‐usual and dry climate scenarios and fisher declines strongest in both dry and wet business‐as‐usual climate scenarios. Alternatively, ringtail occupancy is predicted to increase under all climate scenarios and fox occupancy is predicted to increase or remain stable in warmer and drier climate scenarios. Though our work is focused on the potential effects of changes in temperature and precipitation, these predicted patterns also align with current and predicted changes in the vegetation communities of the Sierra Nevada (Steel et al. 2023). For example, drought and decreasing, or less consistent, snowpack (Asner et al. 2016) has resulted in extensive tree mortality (Goulden and Bales 2019). As a result of extensive tree mortality and shifting climate, extensive and high‐severity wildfires have become more common and resulted in an extensive reduction of mature conifer forests and increases in non‐forested habitat (e.g., shrub, chaparral: Jones et al. 2025). Thus, warming temperatures and decreasing precipitation will continue to impact species' behavioral and physiological thresholds (Bellard et al. 2012), alter resource availability (e.g., Prugh et al. 2009; Calhoun et al. 2025), and reshape the structure and succession of forest communities, ultimately influencing the distribution of forest mesocarnivores across the southern Sierra Nevada.

## 5 Conservation Implications

Climate change and weather anomalies can have asynchronous effects on species of concern, particularly at their range edges. Here, we show that marten, a snow adapted species of conservation concern, appears to decline during atypically dry and warm periods that may result in further declines and potential extirpations if warmer and drier climate conditions persist. Whereas warm adapted or non‐snow dependent species expanded during these same warm and dry periods. In particular, gray fox showed a substantial expansion in occupancy potentially changing carnivore community dynamics as they move into previously unoccupied areas. In our study, long‐term monitoring provided an opportunity to identify shifts in response to novel conditions and track population changes and potential declines for montane mesocarnivores (Tucker et al. 2021; Heiman et al. 2024) and other species in the Sierra Nevada and beyond (Roberts et al. 2019; van den Bosch et al. 2025). Given sparse funding and continued conservation challenges, strong priors and simulations could guide sampling approaches that reduce survey area and costs while maintaining sensitivity to track changes over time (Heiman et al. 2024). Predicted trends in mesocarnivore occupancy assume species' occurrence relationships with spatial and climate conditions will be consistent or static under future climate change scenarios. However, it may be possible that some, or all, of these species could exhibit some spatial plasticity or behavioral plasticity that allows them to persist. Continued monitoring could provide the opportunity not just to track changes in occupancy and population trends but also to identify conditions that could facilitate persistence for species sensitive to change.

## Author Contributions

**Jody Tucker:** conceptualization (equal), data curation (lead), formal analysis (supporting), funding acquisition (lead), investigation (equal), methodology (equal), project administration (lead), resources (lead), supervision (equal), visualization (supporting), writing – original draft (lead). **Marie Martin:** conceptualization (equal), data curation (supporting), formal analysis (lead), funding acquisition (supporting), investigation (equal), methodology (equal), project administration (supporting), resources (supporting), visualization (lead), writing – original draft (supporting), writing – review and editing (lead). **Sean Matthews:** conceptualization (equal), data curation (supporting), formal analysis (supporting), funding acquisition (supporting), investigation (equal), methodology (equal), project administration (equal), resources (supporting), supervision (lead), visualization (supporting), writing – review and editing (supporting). **David Green:** conceptualization (equal), data curation (supporting), formal analysis (lead), investigation (equal), methodology (equal), visualization (supporting), writing – review and editing (equal).

## Funding

This research was funded by the USDA Forest Service Pacific Southwest Region and Rocky Mountain Research Station.

## Conflicts of Interest

The authors declare no conflicts of interest.

## Supporting information


**Figure S1:** Predicted species‐specific changes in occupancy for fishers, gray foxes, martens, and ringtails by elevation band under five climate projection scenarios (Business‐as‐usual emissions [BAU] moderate [CCSM4_rcp85], BAU wet [CNRM_rcp85], BAU dry [MIROC_rcp85], low emissions wet [MPI_rcp45], and low emissions dry [MIROC_rcp45]) and two future time periods (2030–2069, 2070–2099) within the study area in the Sierra Nevada, California, USA.
**Table S1:** Spatial covariates included in detection probability, occupancy (i.e., initial, persistence, colonization), and projection models.
**Table S2:** Annual detection probability for all fisher, gray fox, at track plates and cameras.
**Table S3:** Weekly detection probability at track plates and cameras.
**Table S4:** Detection probability covariate relationships at track plates (β) and cameras (c).
**Table S5:** Species‐specific occupancy covariate relationships for initial occupancy (α), colonization (γ), and persistence (Φ).
**Table S6:** Annual occupancy (i.e., proportion of grid cells occupied) estimates.
**Table S7:** Annual change in occupancy from previous year (lambda) from 2002 to 2015 (below 0 = decrease, > 1 = increase).
**Table S8:** Proportion change in occupancy from baseline (2010) through high snow and drought years at low, mid, and high elevations.

## Data Availability

Code, JAGS model output, and data formatted for JAGS analysis are available at the anonymized repository https://anonymous.4open.science/r/SSN_CarnivoreOccupancy‐2638/.
