# Supplementary material for: Long‐Term Population Monitoring Reveals Changes in Mesocarnivore Occupancy in Response to Severe Drought
Source: Ecol Evol. 2026 Jul 16;16(7):e73960. doi: 10.1002/ece3.73960 (PMC13375444; doi:10.1002/ece3.73960)
Supplement: Supplementary file 1 — Figure S1: Predicted species‐specific changes in occupancy for fishers, gray foxes, martens, and ringtails by elevation band under five climate projection scenarios (Business‐as‐usual emissions [BAU] moderate [CCSM4_rcp85], BAU wet [CNRM_rcp85], BAU dry [MIROC_rcp85], low emissions wet [MPI_rcp45], and low emissions dry [MIROC_rcp45]) and two future time periods (2030–2069, 2070–2099) within the study area in the Sierra Nevada, California, USA. Table S1: Spatial covariates included in detection probability, occupancy (i.e., initial, persistence, colonization), and projection models. Table S2: Annual detection probability for all fisher, gray fox, at track plates and cameras. Table S3: Weekly detection probability at track plates and cameras. Table S4: Detection probability covariate relationships at track plates (β) and cameras (c). Table S5: Species‐specific occupancy covariate relationships for initial occupancy (α), colonization (γ), and persistence (Φ). Table S6: Annual occupancy (i.e., proportion of grid cells occupied) estimates. Table S7: Annual change in occupancy from previous year (lambda) from 2002 to 2015 (below 0 = decrease, > 1 = increase). Table S8: Proportion change in occupancy from baseline (2010) through high snow and drought years at low, mid, and high elevations. [file ECE3-16-e73960-s001.docx]

**Supplementary Material**

**Supplemental Methods**

**Spatial Covariates**

We identified fire perimeters >200 acres using Forest Service Vegetation Burn Severity geospatial data version 18_1 ([www.fs.usda.gov/main/r5/landmanagement/gis](http://www.fs.usda.gov/main/r5/landmanagement/gis)). We clipped and then updated canopy cover data within each fire perimeter from a later LANDFIRE update using the mosaic tool in ArcGIS Desktop (version 10.7.1, ESRI 2019) to replace values in the original LANDFIRE layer with post-fire values.

**Assessing species response to drought**

To assess species response to changes in climate and the 2012-2015 drought, we established a pre-drought baseline occupancy rate defined using occupancy values from before 2011 which represented the final year of “typical” climate conditions within our sampling area. We then calculated change in occupancy for subsequent years compared to this baseline with the winter of 2011-2012 being a high precipitation year (171% of normal) and 2012-2015 being four consecutive years of increasingly severe drought ending in 2015 which saw a record low 5% of average snowpack (OEHHA, 2022). Given sampling efforts occurred in the late summer and fall seasons, conditions in a given sampling year reflected the results of winter conditions starting in the prior year (e.g., 2011 sampling represents conditions after the 2010-11 winter).

Previous research has found significant differences in occupancy rates regionally within the study area, therefore, after modeling we also divided the dataset into regions as defined by Zielinski et al. (2013) comprised of the Sierra National Forest, west slope Sequoia National Forest, and the Kern Plateau. Given the relationship between elevation and snowpack we hypothesized that the drought effects might vary by elevation, therefore we also divided the dataset into elevation bands representing low (914-1,372 m), medium (1,372-2,134 m), and high elevations (2,134-3,048 m).

Supplemental Figures

Figure S1. Predicted species-specific changes in occupancy for fishers, gray foxes, martens, and ringtails by elevation band under five climate projection scenarios (Business-as-usual emissions [BAU] moderate [CCSM4_rcp85], BAU wet [CNRM_rcp85], BAU dry [MIROC_rcp85], low emissions wet [MPI_rcp45], and low emissions dry [MIROC_rcp45]) and two future time periods (2030-2069, 2070-2099) within the study area in the Sierra Nevada, California, USA.

1.
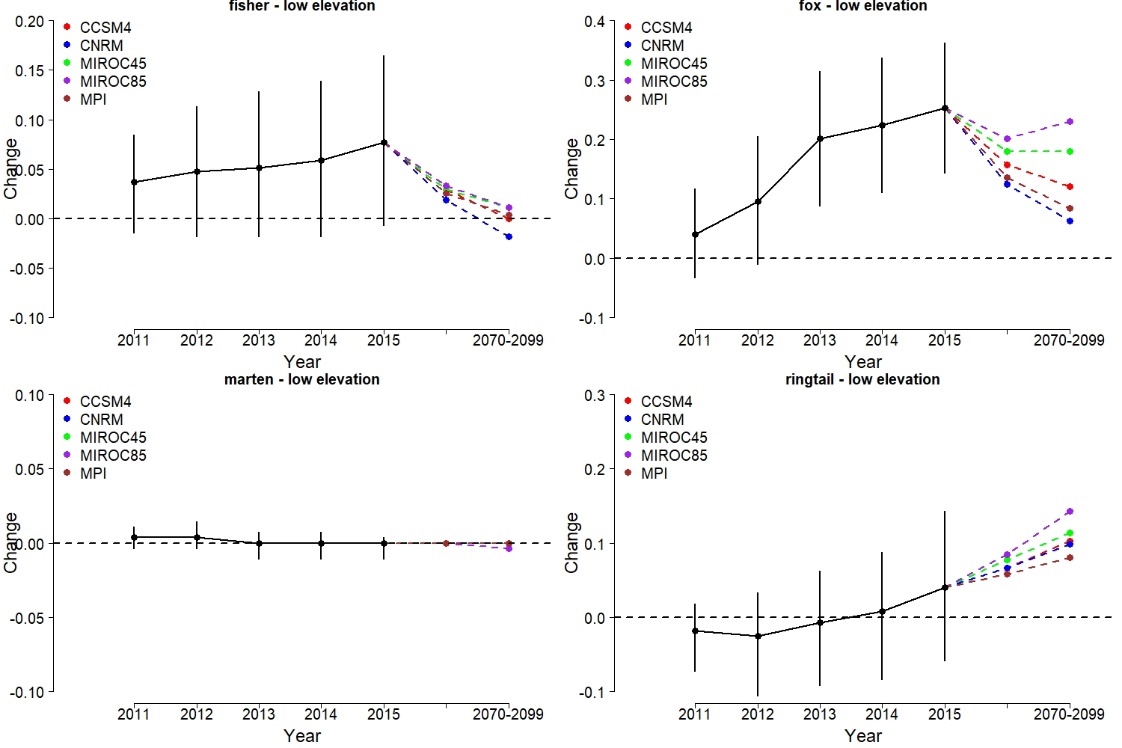
Low elevations
2.
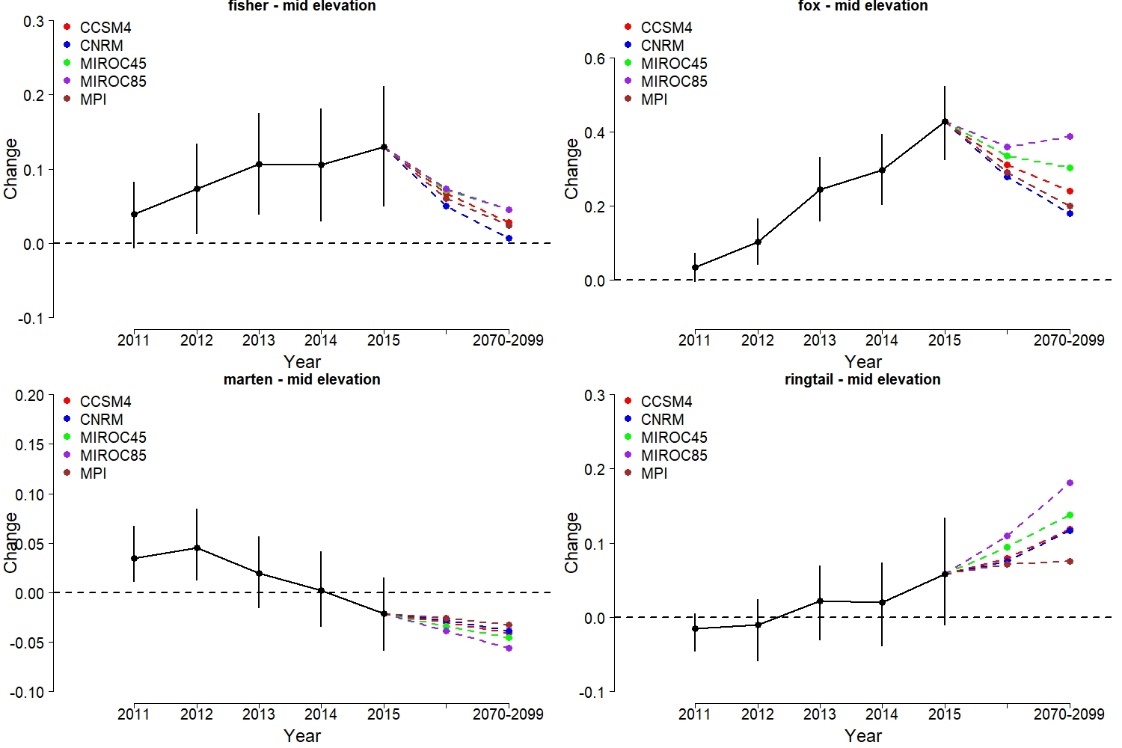
Middle elevations
3. High Elevations


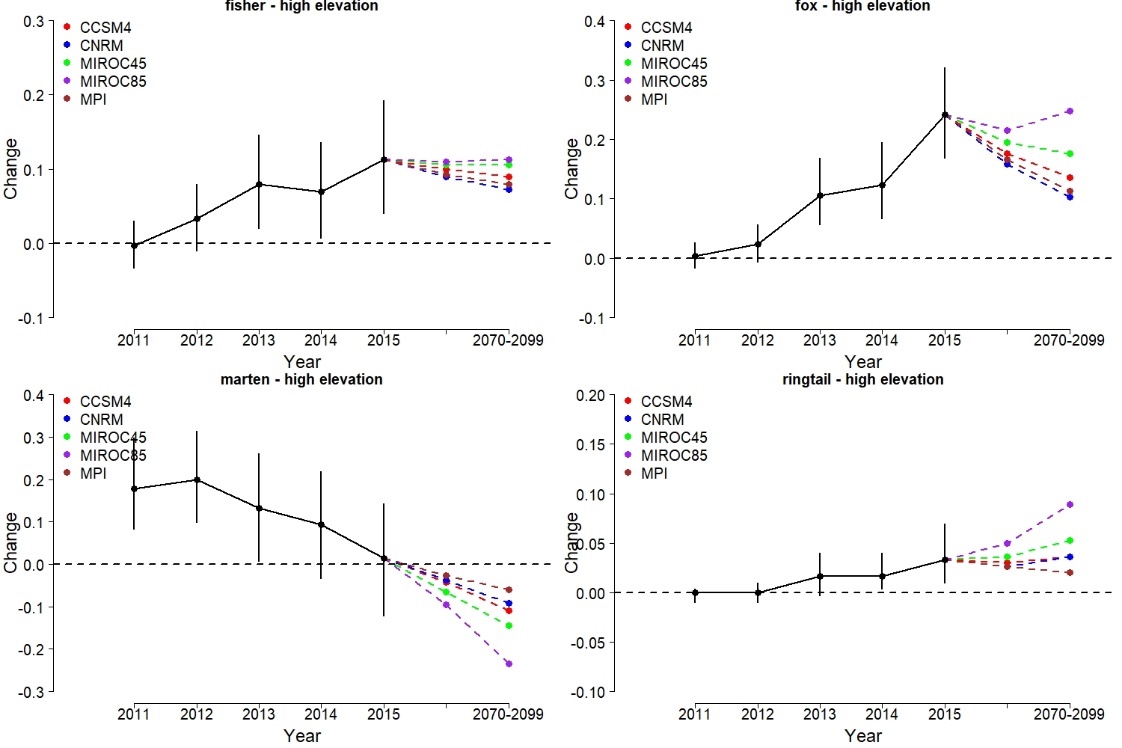


**Supplemental Tables**

Table S1. Spatial covariates included in detection probability, occupancy (i.e., initial, persistence, colonization), and projection models.

| Parameter | Covariate | Source | Unit | Native resolution | Model resolution |
| --- | --- | --- | --- | --- | --- |
| Detection probability | Canopy cover | LANDFIRE | % | 30 m^2^ | 100 m^2^ |
|  | SD canopy cover | LANDFIRE | % | 30 m^2^ | 100 m^2^ |
| Occupancy | Canopy cover | LANDFIRE | % | 30 m^2^ | 6.25 km^2^ |
|  | SD canopy cover | LANDFIRE | % | 30 m^2^ | 6.25 km^2^ |
|  | Snowpack | BCM | mm | 270 m^2^ | 6.25 km^2^ |
|  | Precipitation | BCM | mm | 270 m^2^ | 6.25 km^2^ |
|  | Minimum temperature | BCM | °C | 270 m^2^ | 6.25 km^2^ |
| Projected occupancy | Projected snowpack | BCM | mm | 270 m^2^ | 6.25 km^2^ |
|  | Projected precipitation | BCM | mm | 270 m^2^ | 6.25 km^2^ |
|  | Projected minimum temperature | BCM | °C | 270 m^2^ | 6.25 km^2^ |

Table S2. Annual detection probability for all fisher, gray fox, at track plates and cameras

| Species | Param | Mean | SD | Low | Median | High |
| --- | --- | --- | --- | --- | --- | --- |
| Fisher | det.tra.t_2002_ | 0.25 | 0.03 | 0.18 | 0.25 | 0.31 |
| Fisher | det.tra.t_2003_ | 0.23 | 0.03 | 0.17 | 0.23 | 0.29 |
| Fisher | det.tra.t_2004_ | 0.24 | 0.03 | 0.19 | 0.24 | 0.30 |
| Fisher | det.tra.t_2005_ | 0.32 | 0.03 | 0.26 | 0.32 | 0.38 |
| Fisher | det.tra.t_2006_ | 0.35 | 0.02 | 0.30 | 0.35 | 0.39 |
| Fisher | det.tra.t_2007_ | 0.29 | 0.02 | 0.25 | 0.29 | 0.34 |
| Fisher | det.tra.t_2008_ | 0.36 | 0.03 | 0.30 | 0.36 | 0.41 |
| Fisher | det.tra.t_2009_ | 0.43 | 0.03 | 0.37 | 0.43 | 0.50 |
| Fisher | det.tra.t_2011_ | 0.50 | 0.05 | 0.40 | 0.50 | 0.61 |
| Fisher | det.tra.t_2012_ | 0.54 | 0.06 | 0.43 | 0.54 | 0.66 |
| Fisher | det.tra.t_2013_ | 0.47 | 0.06 | 0.35 | 0.47 | 0.59 |
| Fisher | det.tra.t_2014_ | 0.42 | 0.05 | 0.32 | 0.42 | 0.52 |
| Fisher | det.tra.t_2015_ | 0.47 | 0.05 | 0.37 | 0.47 | 0.56 |
| Fisher | det.cam.t_2011_ | 0.64 | 0.05 | 0.55 | 0.64 | 0.73 |
| Fisher | det.cam.t_2012_ | 0.70 | 0.06 | 0.58 | 0.70 | 0.82 |
| Fisher | det.cam.t_2013_ | 0.64 | 0.05 | 0.54 | 0.64 | 0.75 |
| Fisher | det.cam.t_2014_ | 0.59 | 0.05 | 0.48 | 0.59 | 0.68 |
| Fisher | det.cam.t_2015_ | 0.58 | 0.05 | 0.48 | 0.58 | 0.67 |
| Fox | det.tra.t_2002_ | 0.32 | 0.04 | 0.25 | 0.31 | 0.39 |
| Fox | det.tra.t_2003_ | 0.25 | 0.03 | 0.20 | 0.25 | 0.31 |
| Fox | det.tra.t_2004_ | 0.26 | 0.04 | 0.20 | 0.26 | 0.34 |
| Fox | det.tra.t_2005_ | 0.23 | 0.03 | 0.18 | 0.23 | 0.30 |
| Fox | det.tra.t_2006_ | 0.20 | 0.03 | 0.15 | 0.20 | 0.26 |
| Fox | det.tra.t_2007_ | 0.31 | 0.03 | 0.25 | 0.31 | 0.37 |
| Fox | det.tra.t_2008_ | 0.28 | 0.03 | 0.22 | 0.27 | 0.33 |
| Fox | det.tra.t_2009_ | 0.36 | 0.03 | 0.30 | 0.36 | 0.43 |
| Fox | det.tra.t_2011_ | 0.49 | 0.06 | 0.38 | 0.49 | 0.61 |
| Fox | det.tra.t_2012_ | 0.38 | 0.06 | 0.26 | 0.38 | 0.51 |
| Fox | det.tra.t_2013_ | 0.56 | 0.06 | 0.45 | 0.56 | 0.67 |
| Fox | det.tra.t_2014_ | 0.35 | 0.04 | 0.27 | 0.35 | 0.43 |
| Fox | det.tra.t_2015_ | 0.47 | 0.04 | 0.40 | 0.47 | 0.56 |
| Fox | det.cam.t_2011_ | 0.80 | 0.04 | 0.72 | 0.80 | 0.88 |
| Fox | det.cam.t_2012_ | 0.76 | 0.04 | 0.67 | 0.76 | 0.83 |
| Fox | det.cam.t_2013_ | 0.80 | 0.04 | 0.73 | 0.80 | 0.87 |
| Fox | det.cam.t_2014_ | 0.71 | 0.04 | 0.62 | 0.71 | 0.78 |
| Fox | det.cam.t_2015_ | 0.78 | 0.03 | 0.72 | 0.78 | 0.84 |
| Marten | det.tra.t_2002_ | 0.28 | 0.03 | 0.22 | 0.28 | 0.35 |
| Marten | det.tra.t_2003_ | 0.22 | 0.05 | 0.13 | 0.22 | 0.33 |
| Marten | det.tra.t_2004_ | 0.29 | 0.04 | 0.21 | 0.29 | 0.38 |
| Marten | det.tra.t_2005_ | 0.26 | 0.04 | 0.19 | 0.26 | 0.34 |
| Marten | det.tra.t_2006_ | 0.28 | 0.03 | 0.22 | 0.28 | 0.35 |
| Marten | det.tra.t_2007_ | 0.33 | 0.04 | 0.26 | 0.32 | 0.40 |
| Marten | det.tra.t_2008_ | 0.32 | 0.04 | 0.24 | 0.32 | 0.40 |
| Marten | det.tra.t_2009_ | 0.28 | 0.06 | 0.18 | 0.28 | 0.40 |
| Marten | det.tra.t_2011_ | 0.40 | 0.06 | 0.28 | 0.39 | 0.53 |
| Marten | det.tra.t_2012_ | 0.47 | 0.06 | 0.35 | 0.46 | 0.59 |
| Marten | det.tra.t_2013_ | 0.42 | 0.07 | 0.29 | 0.42 | 0.58 |
| Marten | det.tra.t_2014_ | 0.42 | 0.07 | 0.30 | 0.42 | 0.56 |
| Marten | det.tra.t_2015_ | 0.38 | 0.06 | 0.28 | 0.38 | 0.51 |
| Marten | det.cam.t_2011_ | 0.70 | 0.05 | 0.59 | 0.70 | 0.80 |
| Marten | det.cam.t_2012_ | 0.72 | 0.05 | 0.63 | 0.72 | 0.82 |
| Marten | det.cam.t_2013_ | 0.65 | 0.06 | 0.51 | 0.66 | 0.76 |
| Marten | det.cam.t_2014_ | 0.66 | 0.06 | 0.53 | 0.66 | 0.76 |
| Marten | det.cam.t_2015_ | 0.67 | 0.05 | 0.55 | 0.67 | 0.76 |
| Ringtail | det.tra.t_2002_ | 0.22 | 0.05 | 0.12 | 0.22 | 0.33 |
| Ringtail | det.tra.t_2003_ | 0.21 | 0.05 | 0.12 | 0.20 | 0.30 |
| Ringtail | det.tra.t_2004_ | 0.29 | 0.05 | 0.21 | 0.29 | 0.39 |
| Ringtail | det.tra.t_2005_ | 0.32 | 0.04 | 0.24 | 0.32 | 0.40 |
| Ringtail | det.tra.t_2006_ | 0.31 | 0.03 | 0.25 | 0.31 | 0.38 |
| Ringtail | det.tra.t_2007_ | 0.38 | 0.04 | 0.31 | 0.37 | 0.45 |
| Ringtail | det.tra.t_2008_ | 0.28 | 0.03 | 0.22 | 0.27 | 0.34 |
| Ringtail | det.tra.t_2009_ | 0.34 | 0.03 | 0.27 | 0.34 | 0.41 |
| Ringtail | det.tra.t_2011_ | 0.47 | 0.07 | 0.34 | 0.47 | 0.62 |
| Ringtail | det.tra.t_2012_ | 0.44 | 0.08 | 0.30 | 0.44 | 0.61 |
| Ringtail | det.tra.t_2013_ | 0.45 | 0.07 | 0.32 | 0.44 | 0.60 |
| Ringtail | det.tra.t_2014_ | 0.41 | 0.06 | 0.29 | 0.40 | 0.54 |
| Ringtail | det.tra.t_2015_ | 0.33 | 0.06 | 0.22 | 0.33 | 0.45 |
| Ringtail | det.cam.t_2011_ | 0.67 | 0.06 | 0.55 | 0.67 | 0.79 |
| Ringtail | det.cam.t_2012_ | 0.69 | 0.07 | 0.56 | 0.69 | 0.82 |
| Ringtail | det.cam.t_2013_ | 0.65 | 0.06 | 0.53 | 0.65 | 0.77 |
| Ringtail | det.cam.t_2014_ | 0.62 | 0.07 | 0.47 | 0.62 | 0.74 |
| Ringtail | det.cam.t_2015_ | 0.59 | 0.07 | 0.44 | 0.60 | 0.72 |

Table S3. Weekly detection probability at track plates and cameras

| Species | Param | Mean | SD | Low | Median | High |
| --- | --- | --- | --- | --- | --- | --- |
| Fisher | det.tra.v_2002_ | 0.06 | 0.01 | 0.04 | 0.05 | 0.07 |
| Fisher | det.tra.v_2003_ | 0.05 | 0.01 | 0.04 | 0.05 | 0.07 |
| Fisher | det.tra.v_2004_ | 0.05 | 0.01 | 0.04 | 0.05 | 0.07 |
| Fisher | det.tra.v_2005_ | 0.07 | 0.01 | 0.06 | 0.07 | 0.09 |
| Fisher | det.tra.v_2006_ | 0.08 | 0.01 | 0.07 | 0.08 | 0.10 |
| Fisher | det.tra.v_2007_ | 0.07 | 0.01 | 0.06 | 0.07 | 0.08 |
| Fisher | det.tra.v_2008_ | 0.08 | 0.01 | 0.07 | 0.08 | 0.10 |
| Fisher | det.tra.v_2009_ | 0.11 | 0.01 | 0.09 | 0.11 | 0.13 |
| Fisher | det.tra.v_2011_ | 0.13 | 0.02 | 0.10 | 0.13 | 0.17 |
| Fisher | det.tra.v_2012_ | 0.15 | 0.02 | 0.10 | 0.14 | 0.20 |
| Fisher | det.tra.v_2013_ | 0.12 | 0.02 | 0.08 | 0.12 | 0.16 |
| Fisher | det.tra.v_2014_ | 0.10 | 0.02 | 0.08 | 0.10 | 0.14 |
| Fisher | det.tra.v_2015_ | 0.12 | 0.02 | 0.09 | 0.12 | 0.15 |
| Fisher | det.cam.v_2011_ | 0.19 | 0.02 | 0.15 | 0.18 | 0.23 |
| Fisher | det.cam.v_2012_ | 0.22 | 0.03 | 0.16 | 0.21 | 0.29 |
| Fisher | det.cam.v_2013_ | 0.19 | 0.03 | 0.14 | 0.19 | 0.25 |
| Fisher | det.cam.v_2014_ | 0.16 | 0.02 | 0.12 | 0.16 | 0.21 |
| Fisher | det.cam.v_2015_ | 0.16 | 0.02 | 0.12 | 0.16 | 0.20 |
| Fox | det.tra.v_2002_ | 0.07 | 0.01 | 0.06 | 0.07 | 0.09 |
| Fox | det.tra.v_2003_ | 0.06 | 0.01 | 0.04 | 0.06 | 0.07 |
| Fox | det.tra.v_2004_ | 0.06 | 0.01 | 0.04 | 0.06 | 0.08 |
| Fox | det.tra.v_2005_ | 0.05 | 0.01 | 0.04 | 0.05 | 0.07 |
| Fox | det.tra.v_2006_ | 0.04 | 0.01 | 0.03 | 0.04 | 0.06 |
| Fox | det.tra.v_2007_ | 0.07 | 0.01 | 0.06 | 0.07 | 0.09 |
| Fox | det.tra.v_2008_ | 0.06 | 0.01 | 0.05 | 0.06 | 0.08 |
| Fox | det.tra.v_2009_ | 0.09 | 0.01 | 0.07 | 0.09 | 0.11 |
| Fox | det.tra.v_2011_ | 0.13 | 0.02 | 0.09 | 0.12 | 0.17 |
| Fox | det.tra.v_2012_ | 0.09 | 0.02 | 0.06 | 0.09 | 0.13 |
| Fox | det.tra.v_2013_ | 0.15 | 0.02 | 0.11 | 0.15 | 0.20 |
| Fox | det.tra.v_2014_ | 0.08 | 0.01 | 0.06 | 0.08 | 0.11 |
| Fox | det.tra.v_2015_ | 0.12 | 0.01 | 0.10 | 0.12 | 0.15 |
| Fox | det.cam.v_2011_ | 0.28 | 0.03 | 0.22 | 0.28 | 0.35 |
| Fox | det.cam.v_2012_ | 0.25 | 0.03 | 0.20 | 0.25 | 0.30 |
| Fox | det.cam.v_2013_ | 0.28 | 0.03 | 0.23 | 0.28 | 0.34 |
| Fox | det.cam.v_2014_ | 0.22 | 0.02 | 0.18 | 0.22 | 0.26 |
| Fox | det.cam.v_2015_ | 0.26 | 0.02 | 0.23 | 0.26 | 0.30 |
| Marten | det.tra.v_2002_ | 0.06 | 0.01 | 0.05 | 0.06 | 0.08 |
| Marten | det.tra.v_2003_ | 0.05 | 0.01 | 0.03 | 0.05 | 0.08 |
| Marten | det.tra.v_2004_ | 0.07 | 0.01 | 0.05 | 0.07 | 0.09 |
| Marten | det.tra.v_2005_ | 0.06 | 0.01 | 0.04 | 0.06 | 0.08 |
| Marten | det.tra.v_2006_ | 0.06 | 0.01 | 0.05 | 0.06 | 0.08 |
| Marten | det.tra.v_2007_ | 0.08 | 0.01 | 0.06 | 0.08 | 0.10 |
| Marten | det.tra.v_2008_ | 0.07 | 0.01 | 0.05 | 0.07 | 0.10 |
| Marten | det.tra.v_2009_ | 0.06 | 0.02 | 0.04 | 0.06 | 0.10 |
| Marten | det.tra.v_2011_ | 0.10 | 0.02 | 0.06 | 0.09 | 0.14 |
| Marten | det.tra.v_2012_ | 0.12 | 0.02 | 0.08 | 0.12 | 0.16 |
| Marten | det.tra.v_2013_ | 0.10 | 0.02 | 0.07 | 0.10 | 0.16 |
| Marten | det.tra.v_2014_ | 0.10 | 0.02 | 0.07 | 0.10 | 0.15 |
| Marten | det.tra.v_2015_ | 0.09 | 0.02 | 0.06 | 0.09 | 0.13 |
| Marten | det.cam.v_2011_ | 0.21 | 0.03 | 0.16 | 0.21 | 0.28 |
| Marten | det.cam.v_2012_ | 0.23 | 0.03 | 0.18 | 0.23 | 0.29 |
| Marten | det.cam.v_2013_ | 0.19 | 0.03 | 0.13 | 0.19 | 0.25 |
| Marten | det.cam.v_2014_ | 0.19 | 0.03 | 0.14 | 0.20 | 0.25 |
| Marten | det.cam.v_2015_ | 0.20 | 0.03 | 0.15 | 0.20 | 0.25 |
| Ringtail | det.tra.v_2002_ | 0.05 | 0.01 | 0.03 | 0.05 | 0.08 |
| Ringtail | det.tra.v_2003_ | 0.05 | 0.01 | 0.03 | 0.04 | 0.07 |
| Ringtail | det.tra.v_2004_ | 0.07 | 0.01 | 0.04 | 0.07 | 0.09 |
| Ringtail | det.tra.v_2005_ | 0.07 | 0.01 | 0.05 | 0.07 | 0.10 |
| Ringtail | det.tra.v_2006_ | 0.07 | 0.01 | 0.06 | 0.07 | 0.09 |
| Ringtail | det.tra.v_2007_ | 0.09 | 0.01 | 0.07 | 0.09 | 0.11 |
| Ringtail | det.tra.v_2008_ | 0.06 | 0.01 | 0.05 | 0.06 | 0.08 |
| Ringtail | det.tra.v_2009_ | 0.08 | 0.01 | 0.06 | 0.08 | 0.10 |
| Ringtail | det.tra.v_2011_ | 0.12 | 0.02 | 0.08 | 0.12 | 0.18 |
| Ringtail | det.tra.v_2012_ | 0.11 | 0.03 | 0.07 | 0.11 | 0.17 |
| Ringtail | det.tra.v_2013_ | 0.11 | 0.02 | 0.07 | 0.11 | 0.17 |
| Ringtail | det.tra.v_2014_ | 0.10 | 0.02 | 0.07 | 0.10 | 0.14 |
| Ringtail | det.tra.v_2015_ | 0.08 | 0.02 | 0.05 | 0.08 | 0.11 |
| Ringtail | det.cam.v_2011_ | 0.20 | 0.03 | 0.15 | 0.20 | 0.27 |
| Ringtail | det.cam.v_2012_ | 0.21 | 0.04 | 0.15 | 0.21 | 0.29 |
| Ringtail | det.cam.v_2013_ | 0.19 | 0.03 | 0.14 | 0.19 | 0.25 |
| Ringtail | det.cam.v_2014_ | 0.18 | 0.03 | 0.12 | 0.18 | 0.23 |
| Ringtail | det.cam.v_2015_ | 0.17 | 0.03 | 0.11 | 0.17 | 0.22 |

Table S4. Detection probability covariate relationships at track plates (β) and cameras (c)

| Species | Param | Mean | SD | Low | Median | High |
| --- | --- | --- | --- | --- | --- | --- |
|  | β_1_*_canopycover_* | 0.37 | 0.06 | 0.29 | 0.37 | 0.48 |
|  | β_2_*_sdcanopycover_* | -0.21 | 0.05 | -0.28 | -0.21 | -0.11 |
|  | β_3_*_previousdet_* | 1.38 | 0.08 | 1.27 | 1.38 | 1.53 |
|  | c_1_*_canopycover_* | 0.44 | 0.12 | 0.21 | 0.43 | 0.67 |
| Fisher | c_2_*_sdcanopycover_* | -0.14 | 0.10 | -0.34 | -0.14 | 0.07 |
|  | c_3_*_cammodel1_* | -0.21 | 0.22 | -0.63 | -0.21 | 0.20 |
|  | c_4_*_cammodel2_* | -0.40 | 0.21 | -0.83 | -0.40 | 0.01 |
|  | c5*_previousdet_* | 1.13 | 0.16 | 0.81 | 1.13 | 1.45 |
|  | β_1_*_canopycover_* | -0.41 | 0.04 | -0.49 | -0.41 | -0.32 |
|  | β_2_*_sdcanopycover_* | -0.03 | 0.04 | -0.11 | -0.03 | 0.05 |
|  | β_3_*_previousdet_* | 2.25 | 0.09 | 2.07 | 2.25 | 2.43 |
|  | c_1_*_canopycover_* | -0.38 | 0.07 | -0.51 | -0.38 | -0.25 |
| Fox | c_2_*_sdcanopycover_* | 0.00 | 0.06 | -0.12 | 0.00 | 0.12 |
|  | c_3_*_cammodel1_* | 0.25 | 0.16 | -0.06 | 0.25 | 0.56 |
|  | c_4_*_cammodel2_* | -0.06 | 0.18 | -0.42 | -0.06 | 0.28 |
|  | c5*_previousdet_* | 0.80 | 0.13 | 0.55 | 0.80 | 1.05 |
|  | β_1_*_canopycover_* | 0.61 | 0.10 | 0.41 | 0.61 | 0.80 |
|  | β_2_*_sdcanopycover_* | -0.27 | 0.10 | -0.46 | -0.27 | -0.08 |
|  | β_3_*_previousdet_* | 1.92 | 0.12 | 1.69 | 1.92 | 2.15 |
|  | c_1_*_canopycover_* | 0.18 | 0.16 | -0.13 | 0.18 | 0.51 |
| Marten | c_2_*_sdcanopycover_* | -0.48 | 0.14 | -0.76 | -0.48 | -0.20 |
|  | c_3_*_cammodel1_* | 0.13 | 0.30 | -0.47 | 0.13 | 0.73 |
|  | c_4_*_cammodel2_* | -0.59 | 0.24 | -1.09 | -0.59 | -0.13 |
|  | c5*_previousdet_* | 1.11 | 0.19 | 0.73 | 1.11 | 1.48 |
|  | β_1_*_canopycover_* | 0.08 | 0.09 | -0.10 | 0.08 | 0.26 |
|  | β_2_*_sdcanopycover_* | 0.14 | 0.07 | 0.01 | 0.14 | 0.28 |
|  | β_3_*_previousdet_* | 2.52 | 0.11 | 2.30 | 2.52 | 2.73 |
|  | c_1_*_canopycover_* | 0.18 | 0.15 | -0.12 | 0.17 | 0.47 |
| Ringtail | c_2_*_sdcanopycover_* | 0.21 | 0.12 | -0.01 | 0.21 | 0.44 |
|  | c_3_*_cammodel1_* | -0.19 | 0.30 | -0.78 | -0.19 | 0.40 |
|  | c_4_*_cammodel2_* | -0.63 | 0.28 | -1.20 | -0.62 | -0.08 |
|  | c5*_previousdet_* | 2.09 | 0.21 | 1.68 | 2.09 | 2.51 |

Table S5. Species-specific occupancy covariate relationships for initial occupancy (𝛼), colonization (γ), and persistence (Φ).

| Species | Parameter | Mean | SD | Low | Median | High |
| --- | --- | --- | --- | --- | --- | --- |
|  | 𝛼_1canopycover_ | 1.30 | 0.46 | 0.48 | 1.27 | 2.29 |
|  | 𝛼_2SDcanopycover_ | 0.11 | 0.28 | -0.43 | 0.10 | 0.67 |
|  | 𝛼_3snowpack_ | -0.16 | 0.72 | -1.55 | -0.16 | 1.25 |
|  | 𝛼_4precipitation_ | -2.54 | 0.90 | -4.37 | -2.52 | -0.83 |
|  | 𝛼_5minimumtemp_ | 0.06 | 0.59 | -1.08 | 0.05 | 1.23 |
|  | γ_1canopycover_ | 1.13 | 0.22 | 0.72 | 1.11 | 1.60 |
|  | γ_2SDcanopycover_ | 0.40 | 0.14 | 0.12 | 0.39 | 0.69 |
| Fisher | γ_3snowpack_ | -0.45 | 0.26 | -0.98 | -0.44 | 0.02 |
|  | γ_4precipitation_ | -0.12 | 0.19 | -0.51 | -0.12 | 0.25 |
|  | γ_5minimumtemp_ | -0.09 | 0.21 | -0.49 | -0.09 | 0.31 |
|  | Φ_1canopycover_ | -0.78 | 0.27 | -1.35 | -0.78 | -0.27 |
|  | Φ_2SDcanopycover_ | -0.97 | 0.18 | -1.35 | -0.97 | -0.62 |
|  | Φ_3snowpack_ | 0.06 | 0.33 | -0.55 | 0.04 | 0.73 |
|  | Φ_4minimumtemp_ | -0.24 | 0.22 | -0.68 | -0.24 | 0.18 |
|  | Φ_5precipitation_ | -0.02 | 0.30 | -0.61 | -0.02 | 0.57 |
|  | 𝛼_1canopycover_ | 0.22 | 0.41 | -0.55 | 0.22 | 1.05 |
|  | 𝛼_2SDcanopycover_ | 0.31 | 0.31 | -0.28 | 0.30 | 0.94 |
|  | 𝛼_3snowpack_ | 0.22 | 0.80 | -1.31 | 0.19 | 1.84 |
|  | 𝛼_4precipitation_ | -2.09 | 0.99 | -4.13 | -2.06 | 0.27 |
|  | 𝛼_5minimumtemp_ | 2.08 | 0.87 | 0.62 | 1.99 | 4.02 |
|  | γ_1canopycover_ | -0.03 | 0.16 | -0.34 | -0.03 | 0.28 |
|  | γ_2SDcanopycover_ | 0.17 | 0.14 | -0.11 | 0.17 | 0.46 |
| Gray fox | γ_3snowpack_ | -0.09 | 0.28 | -0.70 | -0.06 | 0.39 |
|  | γ_4precipitation_ | -1.62 | 0.26 | -2.14 | -1.61 | -1.11 |
|  | γ_5minimumtemp_ | 1.27 | 0.23 | 0.83 | 1.27 | 1.73 |
|  | Φ_1canopycover_ | 0.05 | 0.17 | -0.29 | 0.05 | 0.38 |
|  | Φ_2SDcanopycover_ | -0.29 | 0.18 | -0.55 | -0.18 | 0.15 |
|  | Φ_3snowpack_ | -0.24 | 0.34 | -0.87 | -0.25 | 0.47 |
|  | Φ_4minimumtemp_ | -0.41 | 0.25 | -0.91 | -0.41 | 0.06 |
|  | Φ_5precipitation_ | 0.13 | 0.33 | -0.51 | 0.12 | 0.80 |
|  | 𝛼_1canopycover_ | 0.23 | 0.64 | -1.00 | 0.22 | 1.53 |
|  | 𝛼_2SDcanopycover_ | -0.54 | 0.36 | -1.27 | -0.53 | 0.16 |
|  | 𝛼_3snowpack_ | 2.26 | 0.90 | 0.56 | 2.24 | 4.12 |
|  | 𝛼_4precipitation_ | -2.28 | 1.19 | -4.71 | -2.23 | -0.01 |
|  | 𝛼_5minimumtemp_ | 0.43 | 0.79 | -1.18 | 0.43 | 1.97 |
|  | γ_1canopycover_ | -0.43 | 0.32 | -1.06 | -0.43 | 0.19 |
|  | γ_2SDcanopycover_ | -0.69 | 0.24 | -1.18 | -0.69 | -0.21 |
|  | γ_3snowpack_ | 0.37 | 0.28 | -0.19 | 0.37 | 0.89 |
| Marten | γ_4precipitation_ | 0.55 | 0.24 | 0.09 | 0.55 | 1.04 |
|  | γ_5minimumtemp_ | -1.31 | 0.41 | -2.14 | -1.30 | -0.54 |
|  | Φ_1canopycover_ | 0.92 | 0.49 | -0.02 | 0.92 | 1.92 |
|  | Φ_2SDcanopycover_ | 0.11 | 0.32 | -0.54 | 0.12 | 0.74 |
|  | Φ_3snowpack_ | -0.09 | 0.32 | -0.69 | -0.11 | 0.57 |
|  | Φ_4minimumtemp_ | 0.74 | 0.41 | -0.03 | 0.73 | 1.60 |
|  | Φ_5precipitation_ | -1.56 | 0.50 | -2.61 | -1.54 | -0.64 |
|  | 𝛼_1canopycover_ | 0.21 | 1.03 | -2.44 | 0.33 | 1.77 |
|  | 𝛼_2SDcanopycover_ | 0.24 | 0.51 | -0.75 | 0.23 | 1.30 |
|  | 𝛼_3snowpack_ | 0.25 | 1.47 | -2.45 | 0.17 | 3.55 |
|  | 𝛼_4precipitation_ | -2.86 | 1.61 | -6.52 | -2.72 | -0.11 |
|  | 𝛼_5minimumtemp_ | 2.12 | 1.66 | 0.07 | 1.69 | 6.91 |
|  | γ_1canopycover_ | 0.46 | 0.29 | -0.09 | 0.46 | 1.04 |
|  | γ_2SDcanopycover_ | 0.11 | 0.20 | -0.27 | 0.11 | 0.50 |
| Ringtail | γ_3snowpack_ | -0.17 | 0.49 | -1.23 | 0.27 | 0.84 |
|  | γ_4precipitation_ | -0.77 | 0.30 | -1.32 | -0.78 | -0.08 |
|  | γ_5minimumtemp_ | 1.21 | 0.35 | 0.48 | 1.22 | 1.87 |
|  | Φ_1canopycover_ | 1.61 | 0.52 | 0.62 | 1.60 | 2.70 |
|  | Φ_2SDcanopycover_ | 0.50 | 0.33 | -0.14 | 0.50 | 1.17 |
|  | Φ_3snowpack_ | -1.68 | 1.12 | -3.64 | -1.80 | 0.76 |
|  | Φ_4minimumtemp_ | -0.13 | 0.58 | -1.36 | -0.09 | 0.89 |
|  | Φ_5precipitation_ | 0.31 | 0.76 | -1.00 | 0.24 | 1.97 |

Table S6. Annual occupancy (i.e., proportion of grid cells occupied) estimates

| Species | Param | Mean | SD | Low | Median | High |
| --- | --- | --- | --- | --- | --- | --- |
|  | p.occ2002 | 0.35 | 0.05 | 0.26 | 0.35 | 0.46 |
|  | p.occ2003 | 0.32 | 0.03 | 0.26 | 0.32 | 0.40 |
|  | p.occ2004 | 0.32 | 0.03 | 0.27 | 0.32 | 0.38 |
|  | p.occ2005 | 0.29 | 0.03 | 0.24 | 0.29 | 0.34 |
|  | p.occ2006 | 0.27 | 0.02 | 0.22 | 0.27 | 0.32 |
|  | p.occ2007 | 0.29 | 0.02 | 0.25 | 0.29 | 0.34 |
|  | p.occ2008 | 0.28 | 0.02 | 0.24 | 0.28 | 0.33 |
| Fisher | p.occ2009 | 0.27 | 0.02 | 0.23 | 0.27 | 0.32 |
|  | p.occ2010 | 0.27 | 0.02 | 0.22 | 0.27 | 0.31 |
|  | p.occ2011 | 0.28 | 0.02 | 0.24 | 0.28 | 0.33 |
|  | p.occ2012 | 0.31 | 0.02 | 0.27 | 0.31 | 0.36 |
|  | p.occ2013 | 0.34 | 0.03 | 0.29 | 0.34 | 0.39 |
|  | p.occ2014 | 0.34 | 0.03 | 0.28 | 0.33 | 0.39 |
|  | p.occ2015 | 0.36 | 0.03 | 0.31 | 0.36 | 0.42 |
|  | p.occ2002 | 0.41 | 0.04 | 0.32 | 0.41 | 0.50 |
|  | p.occ2003 | 0.36 | 0.03 | 0.30 | 0.36 | 0.42 |
|  | p.occ2004 | 0.37 | 0.02 | 0.33 | 0.37 | 0.42 |
|  | p.occ2005 | 0.28 | 0.03 | 0.24 | 0.28 | 0.34 |
|  | p.occ2006 | 0.24 | 0.03 | 0.19 | 0.24 | 0.29 |
|  | p.occ2007 | 0.34 | 0.02 | 0.30 | 0.34 | 0.39 |
|  | p.occ2008 | 0.35 | 0.02 | 0.31 | 0.35 | 0.40 |
| Fox | p.occ2009 | 0.35 | 0.02 | 0.30 | 0.35 | 0.39 |
|  | p.occ2010 | 0.29 | 0.02 | 0.24 | 0.29 | 0.34 |
|  | p.occ2011 | 0.31 | 0.02 | 0.27 | 0.31 | 0.35 |
|  | p.occ2012 | 0.36 | 0.02 | 0.32 | 0.36 | 0.41 |
|  | p.occ2013 | 0.47 | 0.03 | 0.42 | 0.47 | 0.52 |
|  | p.occ2014 | 0.51 | 0.03 | 0.45 | 0.51 | 0.56 |
|  | p.occ2015 | 0.61 | 0.03 | 0.55 | 0.61 | 0.66 |
|  | p.occ2002 | 0.25 | 0.05 | 0.17 | 0.25 | 0.35 |
|  | p.occ2003 | 0.18 | 0.02 | 0.14 | 0.18 | 0.23 |
|  | p.occ2004 | 0.15 | 0.02 | 0.12 | 0.15 | 0.19 |
|  | p.occ2005 | 0.21 | 0.02 | 0.17 | 0.21 | 0.24 |
|  | p.occ2006 | 0.22 | 0.02 | 0.18 | 0.22 | 0.26 |
|  | p.occ2007 | 0.16 | 0.02 | 0.13 | 0.16 | 0.20 |
|  | p.occ2008 | 0.14 | 0.02 | 0.11 | 0.14 | 0.18 |
| Marten | p.occ2009 | 0.14 | 0.02 | 0.10 | 0.14 | 0.17 |
|  | p.occ2010 | 0.18 | 0.02 | 0.14 | 0.18 | 0.22 |
|  | p.occ2011 | 0.25 | 0.02 | 0.22 | 0.25 | 0.29 |
|  | p.occ2012 | 0.26 | 0.02 | 0.22 | 0.26 | 0.30 |
|  | p.occ2013 | 0.22 | 0.02 | 0.18 | 0.22 | 0.26 |
|  | p.occ2014 | 0.21 | 0.02 | 0.17 | 0.21 | 0.25 |
|  | p.occ2015 | 0.16 | 0.02 | 0.12 | 0.16 | 0.20 |
|  | p.occ2002 | 0.27 | 0.08 | 0.14 | 0.26 | 0.43 |
|  | p.occ2003 | 0.22 | 0.04 | 0.15 | 0.21 | 0.31 |
|  | p.occ2004 | 0.22 | 0.03 | 0.17 | 0.22 | 0.28 |
|  | p.occ2005 | 0.19 | 0.02 | 0.15 | 0.19 | 0.24 |
|  | p.occ2006 | 0.17 | 0.02 | 0.13 | 0.17 | 0.22 |
|  | p.occ2007 | 0.20 | 0.02 | 0.16 | 0.20 | 0.25 |
|  | p.occ2008 | 0.21 | 0.02 | 0.17 | 0.21 | 0.26 |
| Ringtail | p.occ2009 | 0.22 | 0.02 | 0.17 | 0.22 | 0.27 |
|  | p.occ2010 | 0.20 | 0.02 | 0.16 | 0.20 | 0.25 |
|  | p.occ2011 | 0.18 | 0.02 | 0.15 | 0.18 | 0.22 |
|  | p.occ2012 | 0.18 | 0.02 | 0.14 | 0.18 | 0.22 |
|  | p.occ2013 | 0.21 | 0.02 | 0.17 | 0.20 | 0.25 |
|  | p.occ2014 | 0.21 | 0.03 | 0.17 | 0.21 | 0.26 |
|  | p.occ2015 | 0.25 | 0.03 | 0.19 | 0.25 | 0.32 |

Table S7. Annual change in occupancy from previous year (lambda) from 2002 to 2015 (below 0 = decrease, > 1 = increase)

| Species | parameter | mean | sd | 2.50% | 50% | 97.50% |
| --- | --- | --- | --- | --- | --- | --- |
| Fisher | lambda_2003_ | 0.93 | 0.07 | 0.80 | 0.92 | 1.08 |
| Fisher | lambda_2004_ | 0.99 | 0.05 | 0.90 | 0.99 | 1.09 |
| Fisher | lambda_2005_ | 0.91 | 0.04 | 0.83 | 0.91 | 1.00 |
| Fisher | lambda_2006_ | 0.92 | 0.04 | 0.85 | 0.92 | 1.00 |
| Fisher | lambda_2007_ | 1.10 | 0.05 | 1.00 | 1.09 | 1.21 |
| Fisher | lambda_2008_ | 0.97 | 0.03 | 0.90 | 0.97 | 1.04 |
| Fisher | lambda_2009_ | 0.97 | 0.03 | 0.90 | 0.97 | 1.04 |
| Fisher | lambda_2010_ | 0.97 | 0.04 | 0.89 | 0.97 | 1.06 |
| Fisher | lambda_2011_ | 1.07 | 0.05 | 0.97 | 1.07 | 1.18 |
| Fisher | lambda_2012_ | 1.09 | 0.05 | 0.99 | 1.09 | 1.20 |
| Fisher | lambda_2013_ | 1.09 | 0.04 | 1.01 | 1.09 | 1.18 |
| Fisher | lambda_2014_ | 0.99 | 0.05 | 0.90 | 0.99 | 1.08 |
| Fisher | lambda_2015_ | 1.07 | 0.04 | 1.00 | 1.07 | 1.15 |
| Fox | lambda_2003_ | 0.87 | 0.06 | 0.77 | 0.87 | 1.00 |
| Fox | lambda_2004_ | 1.04 | 0.05 | 0.94 | 1.04 | 1.16 |
| Fox | lambda_2005_ | 0.77 | 0.05 | 0.67 | 0.77 | 0.86 |
| Fox | lambda_2006_ | 0.84 | 0.04 | 0.76 | 0.84 | 0.92 |
| Fox | lambda_2007_ | 1.44 | 0.12 | 1.23 | 1.43 | 1.69 |
| Fox | lambda_2008_ | 1.02 | 0.04 | 0.95 | 1.02 | 1.09 |
| Fox | lambda_2009_ | 0.99 | 0.03 | 0.93 | 0.99 | 1.05 |
| Fox | lambda_2010_ | 0.83 | 0.03 | 0.76 | 0.84 | 0.90 |
| Fox | lambda_2011_ | 1.09 | 0.06 | 0.98 | 1.09 | 1.21 |
| Fox | lambda_2012_ | 1.17 | 0.05 | 1.07 | 1.16 | 1.28 |
| Fox | lambda_2013_ | 1.30 | 0.05 | 1.21 | 1.30 | 1.41 |
| Fox | lambda_2014_ | 1.07 | 0.05 | 0.98 | 1.07 | 1.18 |
| Fox | lambda_2015_ | 1.20 | 0.04 | 1.13 | 1.20 | 1.27 |
| Marten | lambda_2003_ | 0.74 | 0.11 | 0.54 | 0.74 | 0.95 |
| Marten | lambda_2004_ | 0.83 | 0.05 | 0.72 | 0.82 | 0.93 |
| Marten | lambda_2005_ | 1.38 | 0.13 | 1.15 | 1.37 | 1.67 |
| Marten | lambda_2006_ | 1.07 | 0.06 | 0.94 | 1.06 | 1.19 |
| Marten | lambda_2007_ | 0.74 | 0.05 | 0.64 | 0.74 | 0.84 |
| Marten | lambda_2008_ | 0.89 | 0.05 | 0.78 | 0.89 | 1.00 |
| Marten | lambda_2009_ | 0.94 | 0.06 | 0.83 | 0.94 | 1.05 |
| Marten | lambda_2010_ | 1.34 | 0.11 | 1.15 | 1.32 | 1.59 |
| Marten | lambda_2011_ | 1.40 | 0.14 | 1.17 | 1.39 | 1.71 |
| Marten | lambda_2012_ | 1.04 | 0.06 | 0.93 | 1.04 | 1.16 |
| Marten | lambda_2013_ | 0.83 | 0.05 | 0.73 | 0.83 | 0.93 |
| Marten | lambda_2014_ | 0.97 | 0.11 | 0.76 | 0.97 | 1.18 |
| Marten | lambda_2015_ | 0.77 | 0.06 | 0.65 | 0.77 | 0.89 |
| Ringtail | lambda_2003_ | 0.86 | 0.14 | 0.61 | 0.85 | 1.14 |
| Ringtail | lambda_2004_ | 1.02 | 0.10 | 0.85 | 1.02 | 1.22 |
| Ringtail | lambda_2005_ | 0.86 | 0.07 | 0.72 | 0.86 | 0.98 |
| Ringtail | lambda_2006_ | 0.91 | 0.06 | 0.79 | 0.91 | 1.03 |
| Ringtail | lambda_2007_ | 1.19 | 0.09 | 1.03 | 1.18 | 1.38 |
| Ringtail | lambda_2008_ | 1.04 | 0.05 | 0.94 | 1.04 | 1.14 |
| Ringtail | lambda_2009_ | 1.03 | 0.04 | 0.95 | 1.03 | 1.12 |
| Ringtail | lambda_2010_ | 0.92 | 0.05 | 0.83 | 0.93 | 1.01 |
| Ringtail | lambda_2011_ | 0.91 | 0.06 | 0.80 | 0.91 | 1.02 |
| Ringtail | lambda_2012_ | 0.98 | 0.05 | 0.88 | 0.98 | 1.09 |
| Ringtail | lambda_2013_ | 1.15 | 0.06 | 1.03 | 1.14 | 1.29 |
| Ringtail | lambda_2014_ | 1.03 | 0.07 | 0.89 | 1.03 | 1.17 |
| Ringtail | lambda_2015_ | 1.20 | 0.06 | 1.08 | 1.20 | 1.33 |

Table S8. Proportion change in occupancy from baseline (2010) through high snow and drought years at low, mid, and high elevations

| Species | parameter | mean | sd | 2.50% | 50% | 97.50% | Elevation |
| --- | --- | --- | --- | --- | --- | --- | --- |
| Fisher | pchange[10,1,1] | 0.04 | 0.03 | -0.01 | 0.04 | 0.08 | Low |
| Fisher | pchange[11,1,1] | 0.05 | 0.03 | -0.02 | 0.05 | 0.11 | Low |
| Fisher | pchange[12,1,1] | 0.05 | 0.04 | -0.02 | 0.05 | 0.13 | Low |
| Fisher | pchange[13,1,1] | 0.06 | 0.04 | -0.02 | 0.06 | 0.14 | Low |
| Fisher | pchange[14,1,1] | 0.08 | 0.04 | -0.01 | 0.08 | 0.16 | Low |
| Fox | pchange[10,2,1] | 0.04 | 0.04 | -0.03 | 0.04 | 0.12 | Low |
| Fox | pchange[11,2,1] | 0.10 | 0.06 | -0.01 | 0.10 | 0.21 | Low |
| Fox | pchange[12,2,1] | 0.20 | 0.06 | 0.09 | 0.20 | 0.32 | Low |
| Fox | pchange[13,2,1] | 0.22 | 0.06 | 0.11 | 0.22 | 0.34 | Low |
| Fox | pchange[14,2,1] | 0.25 | 0.06 | 0.14 | 0.25 | 0.36 | Low |
| Marten | pchange[10,3,1] | 0.00 | 0.00 | 0.00 | 0.00 | 0.01 | Low |
| Marten | pchange[11,3,1] | 0.01 | 0.00 | 0.00 | 0.00 | 0.01 | Low |
| Marten | pchange[12,3,1] | 0.00 | 0.00 | -0.01 | 0.00 | 0.01 | Low |
| Marten | pchange[13,3,1] | 0.00 | 0.00 | -0.01 | 0.00 | 0.01 | Low |
| Marten | pchange[14,3,1] | 0.00 | 0.00 | -0.01 | 0.00 | 0.00 | Low |
| Ringtail | pchange[10,4,1] | -0.02 | 0.02 | -0.07 | -0.02 | 0.02 | Low |
| Ringtail | pchange[11,4,1] | -0.03 | 0.04 | -0.11 | -0.03 | 0.03 | Low |
| Ringtail | pchange[12,4,1] | -0.01 | 0.04 | -0.09 | -0.01 | 0.06 | Low |
| Ringtail | pchange[13,4,1] | 0.01 | 0.04 | -0.08 | 0.01 | 0.09 | Low |
| Ringtail | pchange[14,4,1] | 0.04 | 0.05 | -0.06 | 0.04 | 0.14 | Low |
| Fisher | pchange[10,1,2] | 0.04 | 0.02 | -0.01 | 0.04 | 0.08 | Mid |
| Fisher | pchange[11,1,2] | 0.07 | 0.03 | 0.01 | 0.07 | 0.13 | Mid |
| Fisher | pchange[12,1,2] | 0.11 | 0.03 | 0.04 | 0.11 | 0.17 | Mid |
| Fisher | pchange[13,1,2] | 0.11 | 0.04 | 0.03 | 0.11 | 0.18 | Mid |
| Fisher | pchange[14,1,2] | 0.13 | 0.04 | 0.05 | 0.13 | 0.21 | Mid |
| Fox | pchange[10,2,2] | 0.04 | 0.02 | 0.00 | 0.03 | 0.07 | Mid |
| Fox | pchange[11,2,2] | 0.10 | 0.03 | 0.04 | 0.10 | 0.17 | Mid |
| Fox | pchange[12,2,2] | 0.24 | 0.04 | 0.16 | 0.24 | 0.33 | Mid |
| Fox | pchange[13,2,2] | 0.30 | 0.05 | 0.21 | 0.30 | 0.39 | Mid |
| Fox | pchange[14,2,2] | 0.43 | 0.05 | 0.33 | 0.43 | 0.52 | Mid |
| Marten | pchange[10,3,2] | 0.04 | 0.01 | 0.01 | 0.03 | 0.07 | Mid |
| Marten | pchange[11,3,2] | 0.05 | 0.02 | 0.01 | 0.05 | 0.08 | Mid |
| Marten | pchange[12,3,2] | 0.02 | 0.02 | -0.02 | 0.02 | 0.06 | Mid |
| Marten | pchange[13,3,2] | 0.00 | 0.02 | -0.03 | 0.00 | 0.04 | Mid |
| Marten | pchange[14,3,2] | -0.02 | 0.02 | -0.06 | -0.02 | 0.02 | Mid |
| Ringtail | pchange[10,4,2] | -0.02 | 0.01 | -0.05 | -0.02 | 0.00 | Mid |
| Ringtail | pchange[11,4,2] | -0.01 | 0.02 | -0.06 | -0.01 | 0.02 | Mid |
| Ringtail | pchange[12,4,2] | 0.02 | 0.03 | -0.03 | 0.02 | 0.07 | Mid |
| Ringtail | pchange[13,4,2] | 0.02 | 0.03 | -0.04 | 0.02 | 0.07 | Mid |
| Ringtail | pchange[14,4,2] | 0.06 | 0.04 | -0.01 | 0.06 | 0.13 | Mid |
| Fisher | pchange[10,1,3] | 0.00 | 0.02 | -0.03 | 0.00 | 0.03 | High |
| Fisher | pchange[11,1,3] | 0.03 | 0.02 | -0.01 | 0.03 | 0.08 | High |
| Fisher | pchange[12,1,3] | 0.08 | 0.03 | 0.02 | 0.08 | 0.15 | High |
| Fisher | pchange[13,1,3] | 0.07 | 0.03 | 0.01 | 0.07 | 0.14 | High |
| Fisher | pchange[14,1,3] | 0.11 | 0.04 | 0.04 | 0.11 | 0.19 | High |
| Fox | pchange[10,2,3] | 0.00 | 0.01 | -0.02 | 0.00 | 0.03 | High |
| Fox | pchange[11,2,3] | 0.02 | 0.02 | -0.01 | 0.02 | 0.06 | High |
| Fox | pchange[12,2,3] | 0.11 | 0.03 | 0.06 | 0.11 | 0.17 | High |
| Fox | pchange[13,2,3] | 0.12 | 0.03 | 0.07 | 0.12 | 0.20 | High |
| Fox | pchange[14,2,3] | 0.24 | 0.04 | 0.17 | 0.24 | 0.32 | High |
| Marten | pchange[10,3,3] | 0.18 | 0.06 | 0.08 | 0.18 | 0.30 | High |
| Marten | pchange[11,3,3] | 0.20 | 0.05 | 0.10 | 0.20 | 0.31 | High |
| Marten | pchange[12,3,3] | 0.13 | 0.06 | 0.01 | 0.13 | 0.26 | High |
| Marten | pchange[13,3,3] | 0.09 | 0.06 | -0.03 | 0.09 | 0.22 | High |
| Marten | pchange[14,3,3] | 0.01 | 0.07 | -0.12 | 0.01 | 0.14 | High |
| Ringtail | pchange[10,4,3] | 0.00 | 0.00 | -0.01 | 0.00 | 0.00 | High |
| Ringtail | pchange[11,4,3] | 0.00 | 0.01 | -0.01 | 0.00 | 0.01 | High |
| Ringtail | pchange[12,4,3] | 0.02 | 0.01 | 0.00 | 0.02 | 0.04 | High |
| Ringtail | pchange[13,4,3] | 0.02 | 0.01 | 0.00 | 0.02 | 0.04 | High |
| Ringtail | pchange[14,4,3] | 0.04 | 0.02 | 0.01 | 0.03 | 0.07 | High |
